# Supplementary material for: Widespread Post-transcriptional Attenuation of Genomic Copy-Number Variation in Cancer
Source: Cell Syst. 2017 Oct 25;5(4):386–398.e4. doi: 10.1016/j.cels.2017.08.013 (PMC5660600; doi:10.1016/j.cels.2017.08.013)
Supplement: Document S1. Figures S1–S4 [file mmc1.pdf]

Cell Systems, Volume *5*

Supplemental Information

Widespread Post-transcriptional Attenuation of Genomic Copy-Number Variation in Cancer

Emanuel Gonçalves, Athanassios Fragoulis, Luz Garcia-Alonso, Thorsten Cramer, Julio Saez-Rodriguez, and Pedro Beltrao

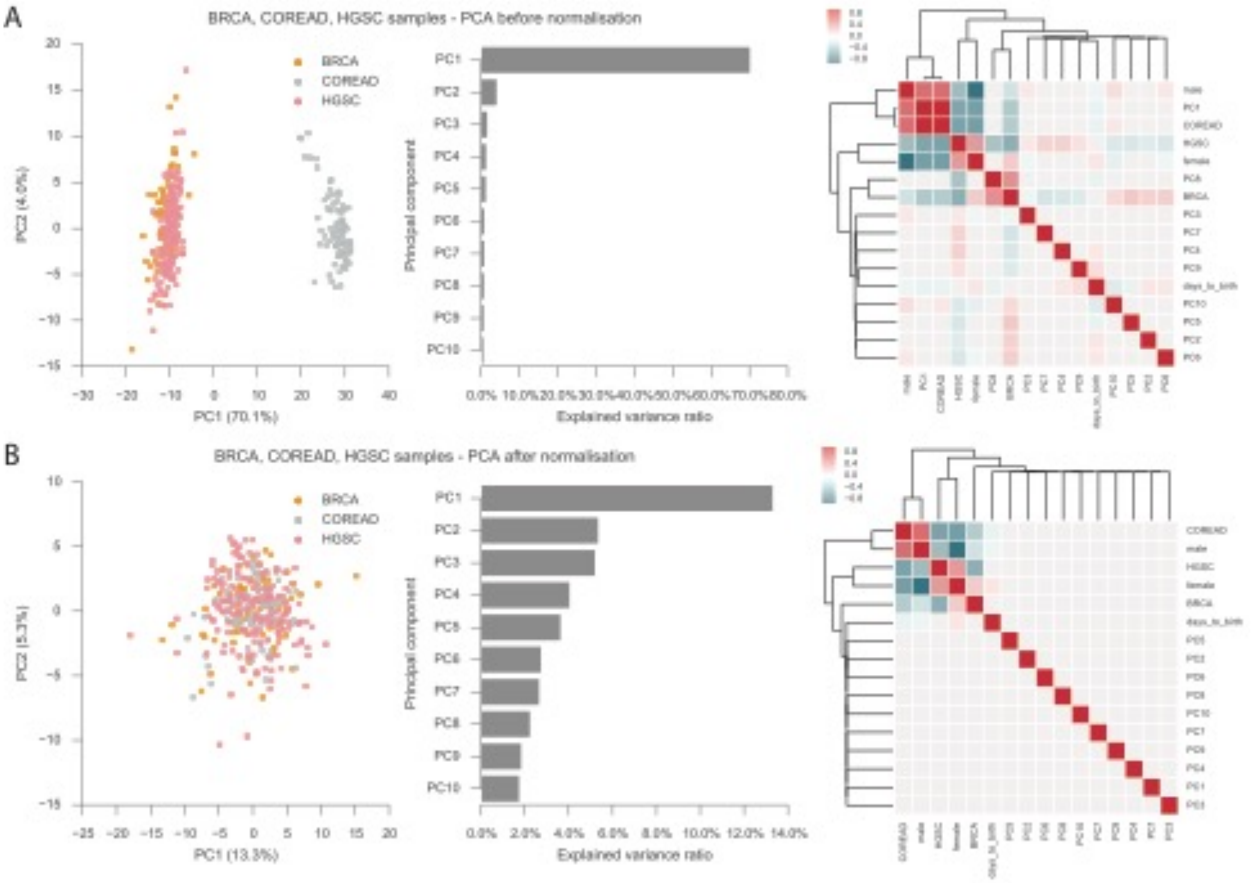

Supplementary Figure 1. Related to Figure 1. Proteomics data-sets PCA analysis using proteins consistently measured across all the samples and pearson correlation coefficient between the first 10 principal components and the possible confounding factors, i.e. age, tumour type and gender. A) Analysis performed on the original proteomics data-sets. B) Analysis performed on the proteomics data-set after the confounding factors were regressed-out.

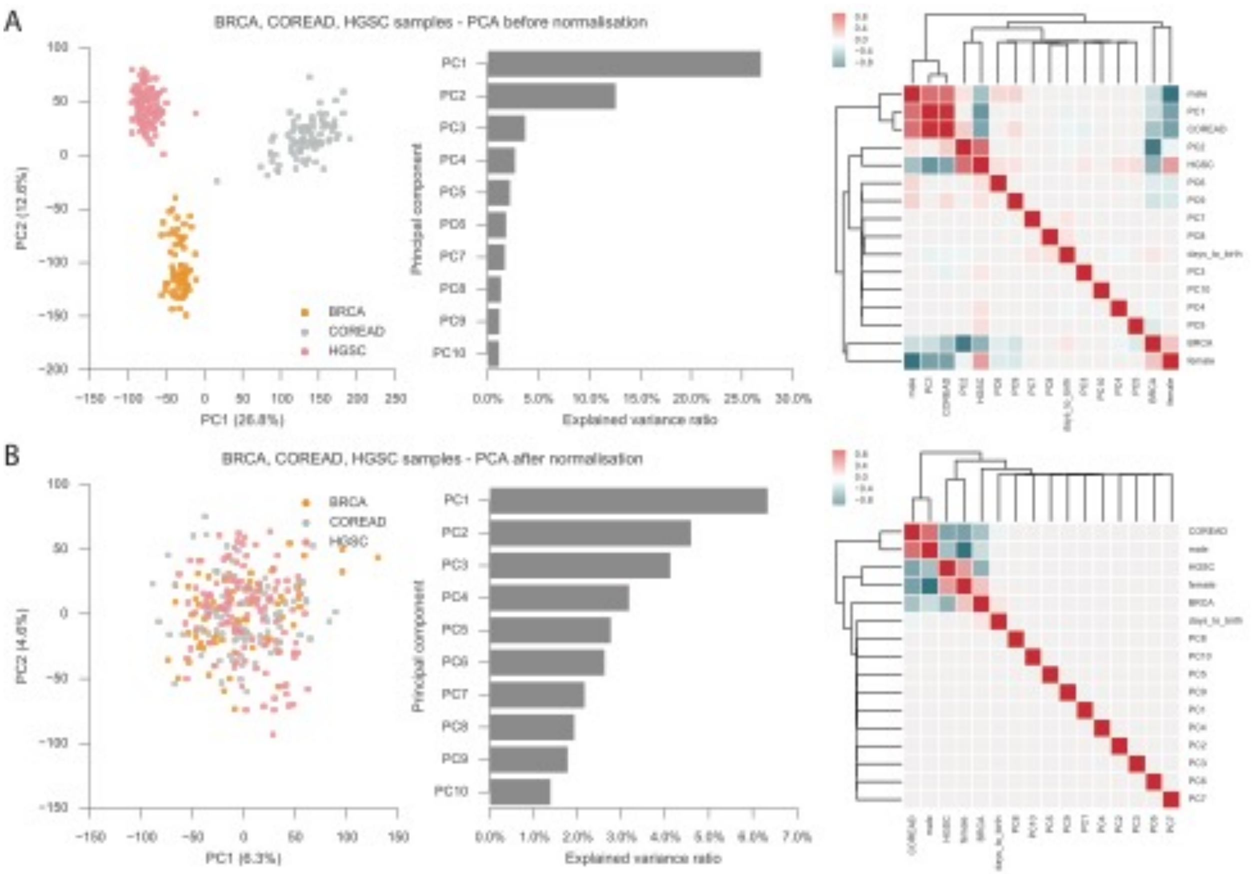

Supplementary Figure 2. Related to Figure 1. Transcriptomics data-sets PCA analysis and pearson correlation coefficient between the first 10 principal components and the possible confounding factors, i.e. age, tumour type and gender. A) Analysis performed on the voom transformed transcriptomics measurements. B) Analysis performed on the transcriptomics data-set after the confounding factors were regressed-out.

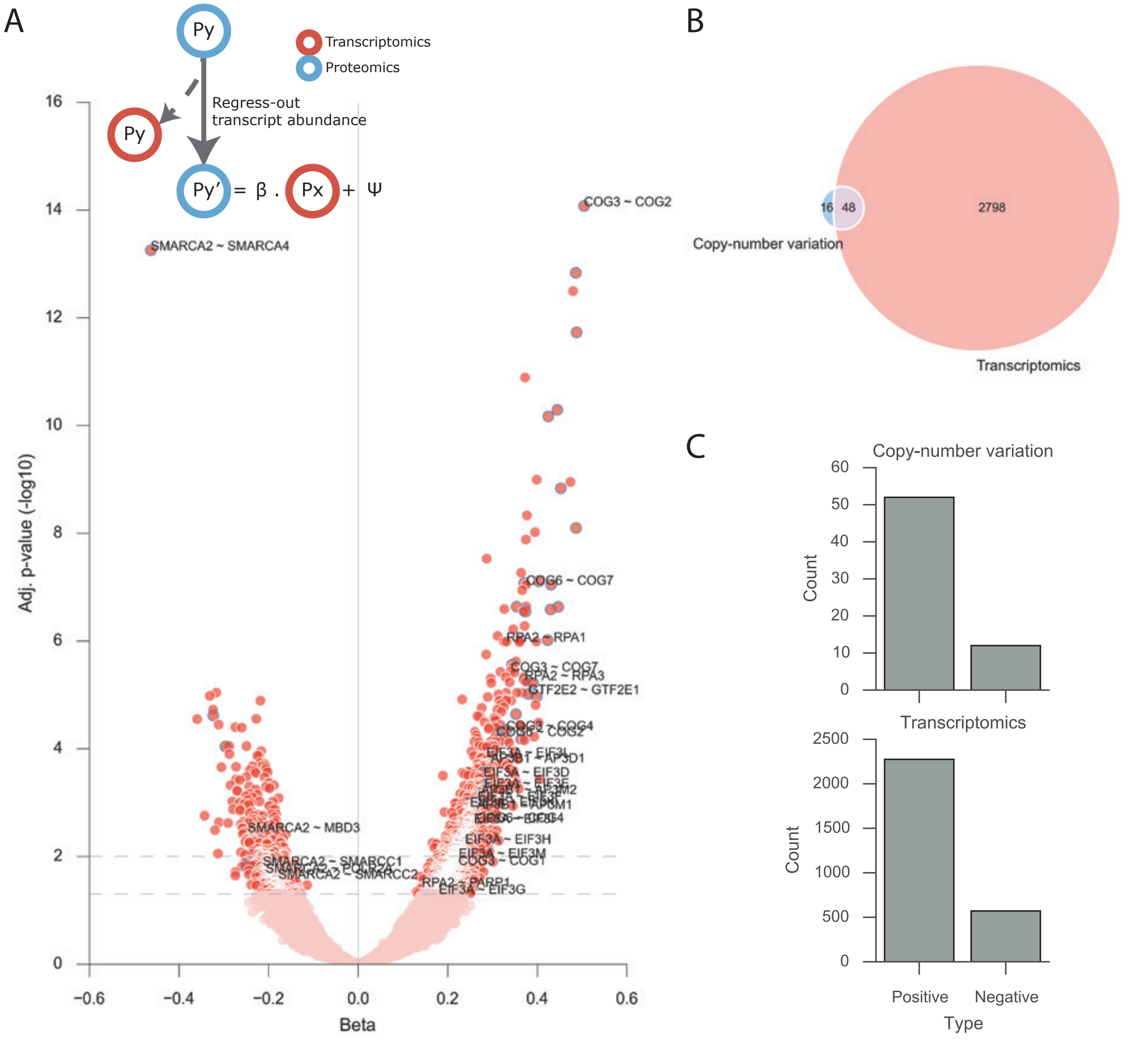

Supplementary Figure 3. Related to Figure 3. Protein complexes regulatory interactions identified using transcriptomics of the putative regulatory protein (Px). A) Volcano plot representing the effect size on the x axis and FDR adjusted p-value on the y axis. Diagram representing the linear model used to perform the associations. B) Overlap between the significant regulatory associations found using the copy-number variation and transcriptomics of the Px proteins. C) Number of significant associations with a Positive or Negative effect size.

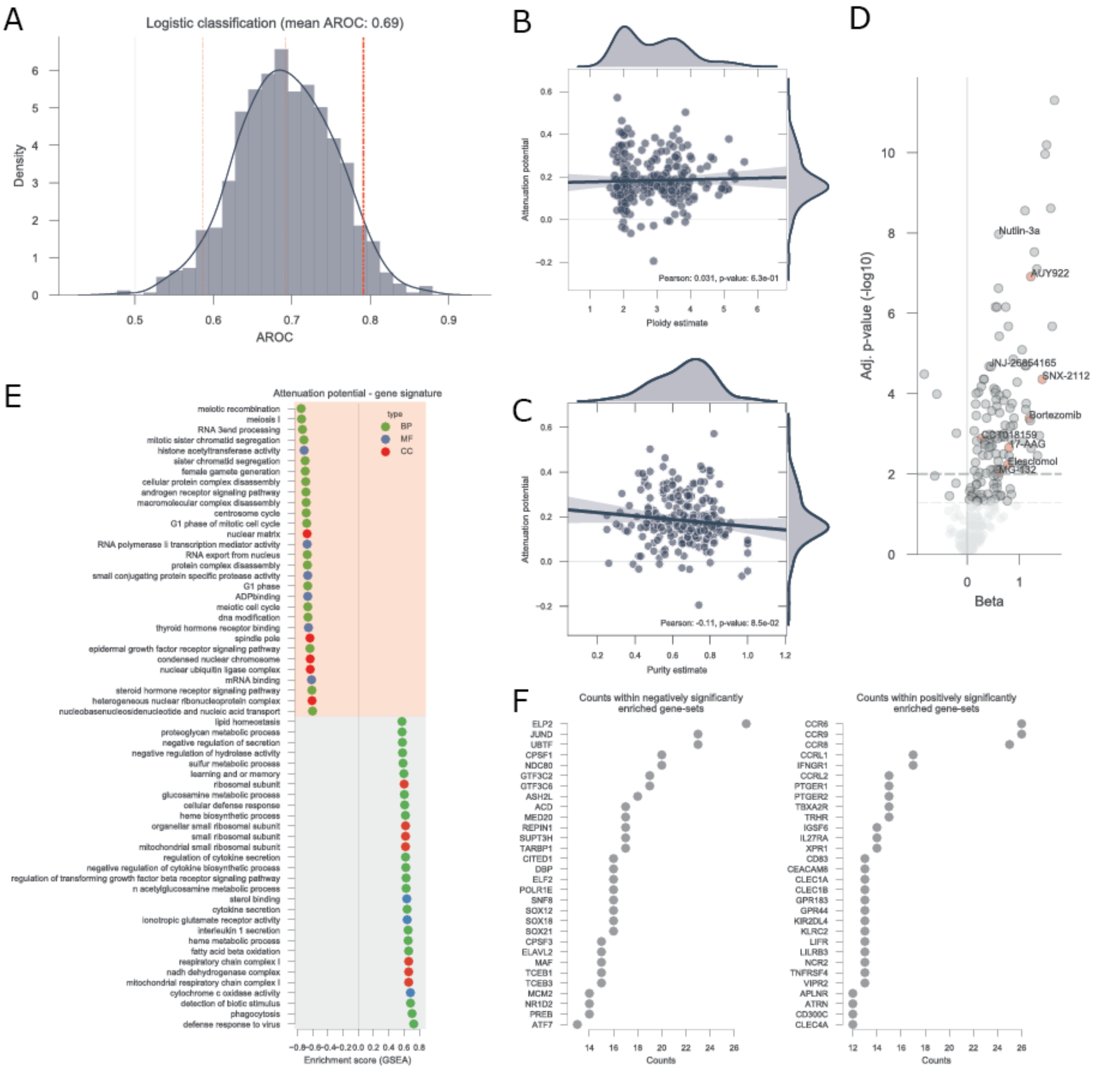

Supplementary Figure 4. Related to Figure 7. Tumour and cell lines samples attenuation potential analysis. A) Benchmark of the gene-signature protein attenuation potential of the tumour samples using a logistic classification model. B) & C) Tumour samples attenuation potential correlation with ploidy and purity, respectively. D) Volcano plot of the drug response associations performed in the cell line panel using the cell lines putative attenuation potential as the predictive feature. Significant associations (FDR < 5%) of chaperone and proteasome inhibitors are labelled and marked in red. Ubiquitin-protein ligase MDM2 inhibitors are labelled. E) GO terms enrichment analysis of the protein attenuation gene-expression signature. Top 30 positive and negative significantly enriched GO terms are shown. Red background denotes GO terms enriched for genes correlating positively with the protein attenuation potential, blue denotes enrichments for negative correlations. F) Shows the genes more frequently present in the significantly enriched gene-signatures. Top 30 most frequent genes are shown for the negative (left) and positive (right) enriched gene-sets.
