# Supplementary material for: Widespread Post-transcriptional Attenuation of Genomic Copy-Number Variation in Cancer
Source: Cell Syst. 2017 Oct 25;5(4):386–398.e4. doi: 10.1016/j.cels.2017.08.013 (PMC5660600; doi:10.1016/j.cels.2017.08.013)
Supplement: Document S2. Article plus Supplemental Information [file mmc6.pdf]

Cell Systems

Article

Widespread Post-transcriptional Attenuation of Genomic Copy-Number Variation in Cancer

Graphical Abstract

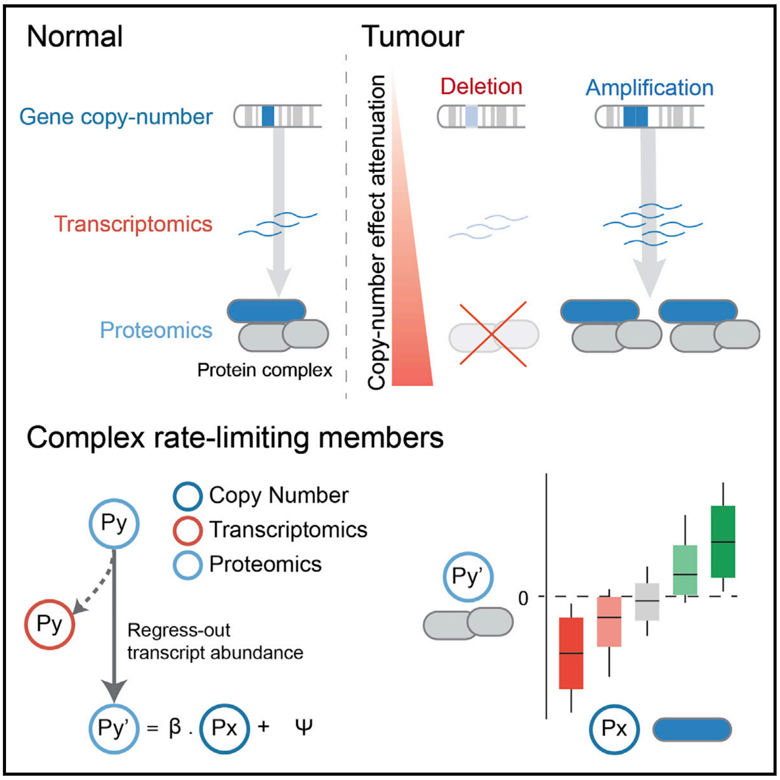

Authors

Emanuel Gonçalves,
Athanassios Fragoulis,
Luz Garcia-Alonso, Thorsten Cramer,
Julio Saez-Rodriguez, Pedro Beltrao

Correspondence

saezrodriguez@gmail.com (J.S.-R.),
pedrobeltrao@ebi.ac.uk (P.B.)

In Brief

Post-transcriptional regulation, likely via control of protein degradation, can attenuate copy-number alterations in tumors for at least 23% of measured proteins. This effect is enriched in protein complexes, with some complex members acting as rate-limiting factors for complex formation.

Highlights

- 23%–33% of the proteins have copy-number changes attenuated, likely via degradation
- Protein complex members are more co-regulated at protein level than mRNA level
- Systematic identification of rate-limiting members for the assembly of the complex
- AP3B1, GTF2E2, and GTF2E1 are limiting members of their complexes

Gonçalves et al., 2017, Cell Systems *5*, 386–398
October 25, 2017 © 2017 The Authors. Published by Elsevier Inc.
https://doi.org/10.1016/j.cels.2017.08.013

OPEN ACCESS
CellPress

Cell Systems
Article

Widespread Post-transcriptional Attenuation of Genomic Copy-Number Variation in Cancer

Emanuel Gonçalves,[1] Athanassios Fragoulis,[3] Luz Garcia-Alonso,[1] Thorsten Cramer,[3,4,5] Julio Saez-Rodriguez,[1,2,*] and Pedro Beltrao[1,6,*]
[1]European Molecular Biology Laboratory, European Bioinformatics Institute (EMBL-EBI), Wellcome Genome Campus, Cambridge CB10 1SD, UK
[2]RWTH Aachen University, Faculty of Medicine, Joint Research Centre for Computational Biomedicine, 52057 Aachen, Germany
[3]Molecular Tumor Biology, Department of General, Visceral and Transplantation Surgery, RWTH University Hospital, Pauwelsstraße 30, 52074 Aachen, Germany
[4]NUTRIM School of Nutrition and Translational Research in Metabolism, Maastricht University, Maastricht, the Netherlands
[5]ESCAM – European Surgery Center Aachen Maastricht, Germany and the Netherlands
[6]Lead Contact
*Correspondence: saezrodriguez@gmail.com (J.S.-R.), pedrobeltrao@ebi.ac.uk (P.B.)
https://doi.org/10.1016/j.cels.2017.08.013

SUMMARY

Copy-number variations (CNVs) are ubiquitous in cancer and often act as driver events, but the effects of CNVs on the proteome of tumors are poorly understood. Here, we analyze recently published genomics, transcriptomics, and proteomics datasets made available by CPTAC and TCGA consortia on 282 breast, ovarian, and colorectal tumor samples to investigate the impact of CNVs in the proteomes of these cells. We found that CNVs are buffered by post-transcriptional regulation in 23%–33% of proteins that are significantly enriched in protein complex members. Our analyses show that complex subunits are highly co-regulated, and some act as rate-limiting steps of complex assembly, as their depletion induces decreased abundance of other complex members. We identified 48 such rate-limiting interactions and experimentally confirmed our predictions on the interactions of AP3B1 with AP3M1 and GTF2E2 with GTF2E1. This study highlights the importance of post-transcriptional mechanisms in cancer that allow cells to cope with their altered genomes.

INTRODUCTION

Cancer development is driven by the acquisition of somatic genetic variation that includes point mutations, copy-number variations (CNVs), and large chromosome rearrangements or duplications (i.e., aneuploidy) (Beroukhim et al., 2010). These events can result in a fitness advantage and cancer progression, but they are most often detrimental to cellular fitness. While somatic gene amplification of key oncogenes such as MYCN, AKT2, ERBB2, and others (Santarius et al., 2010) can drive cancer development, germline CNVs are rare and are under negative selection (Itsara et al., 2009). Gene amplifications and other CNVs are thought to be detrimental due to changes in gene expression that cause an imbalance to the cell. In females, one of the two X chromosomes is inactivated by a specialized RNA-based silencing mechanism (Avner and Heard, 2001; Lyon, 1961), but such a mechanism does not exist for gene-dosage imbalances in the autosomal chromosomes. Protein and mRNA abundance measurements in models of aneuploidy in yeast and human cells have shown that most autosomal gene duplications are propagated to the protein level, with the notable exception of protein complex subunits that showed attenuated (i.e., less than expected) changes in protein abundance (Dephoure et al., 2014; Stingele et al., 2012). In yeast aneuploid strains, the discrepancy between gene copy-number and protein abundance has been shown to be mostly due to control of protein abundance by degradation (Dephoure et al., 2014). For protein complexes in particular, this observation fits with a model where subunits are degraded when free from the complex (Abovich et al., 1985). Given that not all subunits were observed to be attenuated, it has been hypothesized that these non-attenuated subunits could act as scaffolding proteins or be rate-limiting for the assembly of the complex (Dephoure et al., 2014). In addition, duplicated chromosomes have been shown to cause global stress responses that include cell-cycle and metabolic defects and proteotoxic stress among others (Tang and Amon, 2013). While somatic CNVs are known to be drivers of cancer development, and that aneuploidy is a common feature of tumor cells, the impact of gene-dosage changes on the proteome of cancer cells has yet to be studied. We therefore decided to study the extent by which changes in gene copy number are propagated to protein abundance in cancer patient samples, as well as the potential mechanisms underlying the attenuation of protein abundance changes.

In this study, we investigated the implications of CNVs on the proteome of tumors by taking advantage of the comprehensive datasets made available by The Cancer Genome Atlas (TCGA) and the Clinical Proteomic Tumor Analysis Consortium (CPTAC), consortia comprising copy-number, transcript, and protein measurements for hundreds of tumors

 © 2017 The Authors. Published by Elsevier Inc.
This is an open access article under the CC BY license (http://creativecommons.org/licenses/by/4.0/).

(Cancer Genome Atlas Network, 2012a, 2012b; Cancer Genome Atlas Research Network, 2011; Mertins et al., 2016; Zhang et al., 2014, 2016). These data revealed that CNVs are often propagated to the protein level, although we observed that post-transcriptional mechanisms attenuate this impact in 23%–33% of the measured proteins. Protein complexes were notably attenuated and showed strong protein abundance co-regulation across samples. Not all complex subunits are attenuated, with some acting as potential rate-limiting factors for complex assembly. Here we identified 48 regulatory interactions whereby the abundance of one of the subunits can modulate the abundance of other complex members. We experimentally assessed the role of AP3B1 and GTF2E2 as potential rate-limiting subunits through knockdown experiments. In addition, ranking the samples by their potential to attenuate gene-dosage effects identified putative mechanisms involved in autosomal gene-dosage compensation. Finally, a gene expression signature of attenuation potential was found to be associated with drugs targeting chaperones, the proteasome, and the E3 ligase murine double minute 2 (MDM2). Using 282 tumor samples we revealed the widespread importance of post-transcriptional mechanisms to ameliorate the impact of CNVs in cancer cells.

RESULTS

Tumor Pan-cancer Proteomics Reveals Attenuation of Copy-Number Alterations in Protein Complex Subunits

To study the implication of gene-dosage changes on the proteome of cancer cells we compiled and standardized existing datasets made available by the TCGA and CPTAC consortia, comprising three different cancer types: breast (BRCA) (Cancer Genome Atlas Network, 2012b; Mertins et al., 2016), high-grade serous ovarian (HGSC) (Cancer Genome Atlas Research Network, 2011; Zhang et al., 2016), and colon and rectal (COREAD) (Cancer Genome Atlas Network, 2012a; Zhang et al., 2016) (Figure 1A). These datasets provide molecular characterization of gene CNVs, gene expression, and protein abundance of solid tumor samples of 282 patients for which clinical information is also available (Figure 1A, Table S1).

Current methods can reliably measure the complete expressed transcriptome, but measuring the total proteome is still a challenge with current techniques only providing partial snapshots (Nagaraj et al., 2011). Thus, we quantified the fraction of expressed transcripts measured in the proteomics experiments in each tumor sample (Figure 1B) (see the STAR Methods). COREAD samples displayed the lowest average coverage of the expressed transcriptome (22.3%) compared with the coverage measured for the HGSC (42.0%) and BRCA (56.1%) samples. The proteomics experiments were not conducted using the same methodologies, and therefore it is crucial to take into consideration potential confounding effects. In particular, the COREAD (Zhang et al., 2014) quantifications were done with a label-free approach, while the HGSC and BRCA were quantified using isobaric labeling (Mertins et al., 2016; Zhang et al., 2016). To ensure comparable measurements among datasets we removed confounding and systematic effects from the proteomics and transcriptomics, by regressing-out batch effects associated with experimental technologies used, patient gender and age, and tumor type (see the STAR Methods). The associations between these possible confounding factors and the principal components were completely removed after correction (Figures S1 and S2).

Having assembled this compendium of datasets we then set out to understand the implication of CNV events in the expression of the proteome (Figure 1C). For each gene/protein we calculated, across all samples, the agreement between the CNVs and transcriptomics and the CNVs and proteomics using the Pearson correlation coefficient (Figure 1D). Transcript abundance is, on average, well correlated with gene CNV changes (median Pearson's $r = 0.43$), and this contrasts with the significant decrease (Welch's t test p value $< 1 \times 10^{-4}$) of agreement of CNVs with protein abundance (median Pearson's $r = 0.20$) (Figure 1D; Table S2). We hypothesize that, as transcription is intermediate between the copy-number alterations and protein abundance, it sets the maximum possible agreement between both. Then, using a Gaussian mixture model, we defined as attenuated proteins those that have a lower agreement between CNVs and protein abundance than expected by their CNV to gene expression correlation (see the STAR Methods). In these samples we found that, by this definition 1,496–2,119 proteins are significantly attenuated, corresponding to 23%–33% of all genes with available measurements (6,418). This result shows that a significant fraction of the proteome undergoes gene-dosage balancing. In addition, this group of attenuated proteins highlights the complexity of the regulation of protein abundance, hinting at constraints that control protein translation or degradation rates.

To understand the biological processes that are affected by this attenuation we performed an unbiased enrichment analysis using gene ontology terms (Ashburner et al., 2000; Subramanian et al., 2005; The Gene Ontology Consortium, 2015) (Figure 2A) (see the STAR Methods). The enrichment analysis revealed that proteins involved in complexes and modules of functionally interacting proteins displayed a significant agreement with the copy-number measurements at the transcript level, but this agreement is generally lost at the protein level (Figure 2B). This recapitulates previous findings in models of aneuploidy in yeast (Dephoure et al., 2014) and human cell lines (Stingele et al., 2012), showing that these observations generalize from the aneuploidy models to the hundreds of patient tumor samples studied here. To validate the generality of the set of attenuated proteins, we confirmed that these are also recapitulated in independent proteomic cell line panels of triple-negative breast cancer and ovarian cancer (Coscia et al., 2016; Lawrence et al., 2015) (Figure 2C). To test if degradation plays a role in the attenuation observed in human cells, we used publicly available data on changes in protein ubiquitination after proteasome inhibition as markers of degradation (Kim et al., 2011). We observed that proteins defined as attenuated in our study show a faster increase in ubiquitination after proteasome inhibition than other proteins (Figure 2D), suggesting that degradation plays a key role in the attenuation. These results suggest that the abundance of protein subunits of large stable protein complexes are under active control to maintain their co-regulation, possibly to guarantee the stability and formation of the associations or prevent

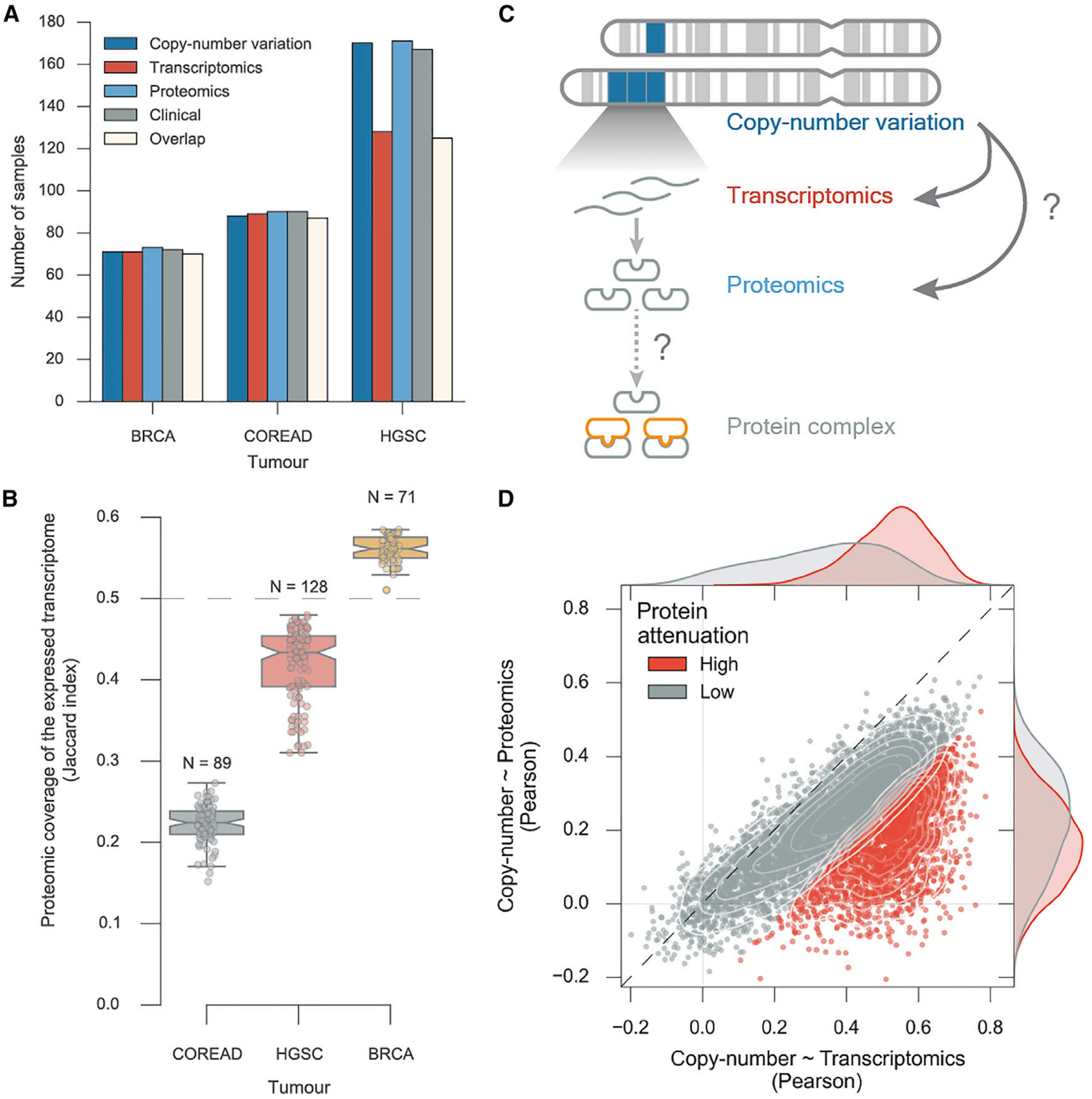

Figure 1. Pan-cancer Effects of Copy-Number Variation on Transcript and Protein Abundances

(A) Overview of the number of samples used in this study overlapping with the proteomics measurements for each tumor type.

(B) Proteomics coverage of the expressed transcripts in each sample and for each tumor type.

(C) Diagram depicting the implication of copy-number alterations along the central dogma of biology.

(D) Each dot in the scatterplot represents a transcript/protein. The x axis represents the Pearson correlation coefficient between copy-number variation and transcriptomics, and the y axis the Pearson correlation between copy-number variation and proteomics. A Gaussian mixture model with two mixture components was used to identify proteins with high attenuation levels (colored in red).

the accumulation of free subunits that might be prone to aggregate.

Proteomic Correlation Analysis Uncovers Strong Co-regulation of Protein Complexes

To test the hypothesis that the attenuation of members serves to tune the stoichiometry of all complex members, we performed protein-protein correlation analysis using the proteomics measurements and compared this with gene expression-based correlations (Figure 3A). We performed all possible pairwise correlation of protein abundance for all the 6,434 proteins measured in at least 50% of the samples across the three different tumor types (see the STAR Methods). Consistently, proteins within the same complexes display coordinated changes of abundance across samples (Figure 3A). Then, we assessed if this co-regulation effect is ubiquitous in a curated set of human protein complexes from the CORUM database (Ruepp et al., 2010). Pairs of proteins present together in a complex display a degree of co-regulation (mean Pearson's r = 0.25) that is significantly higher than that observed for random pairs (mean Pearson's r = 0). We also assessed if this co-regulation was visible at the transcript level, and, while there is a significant increase over random associations (mean Pearson's r = 0.15), this correlation is significantly lower than the one seen at the protein level (Figure 3B). Protein

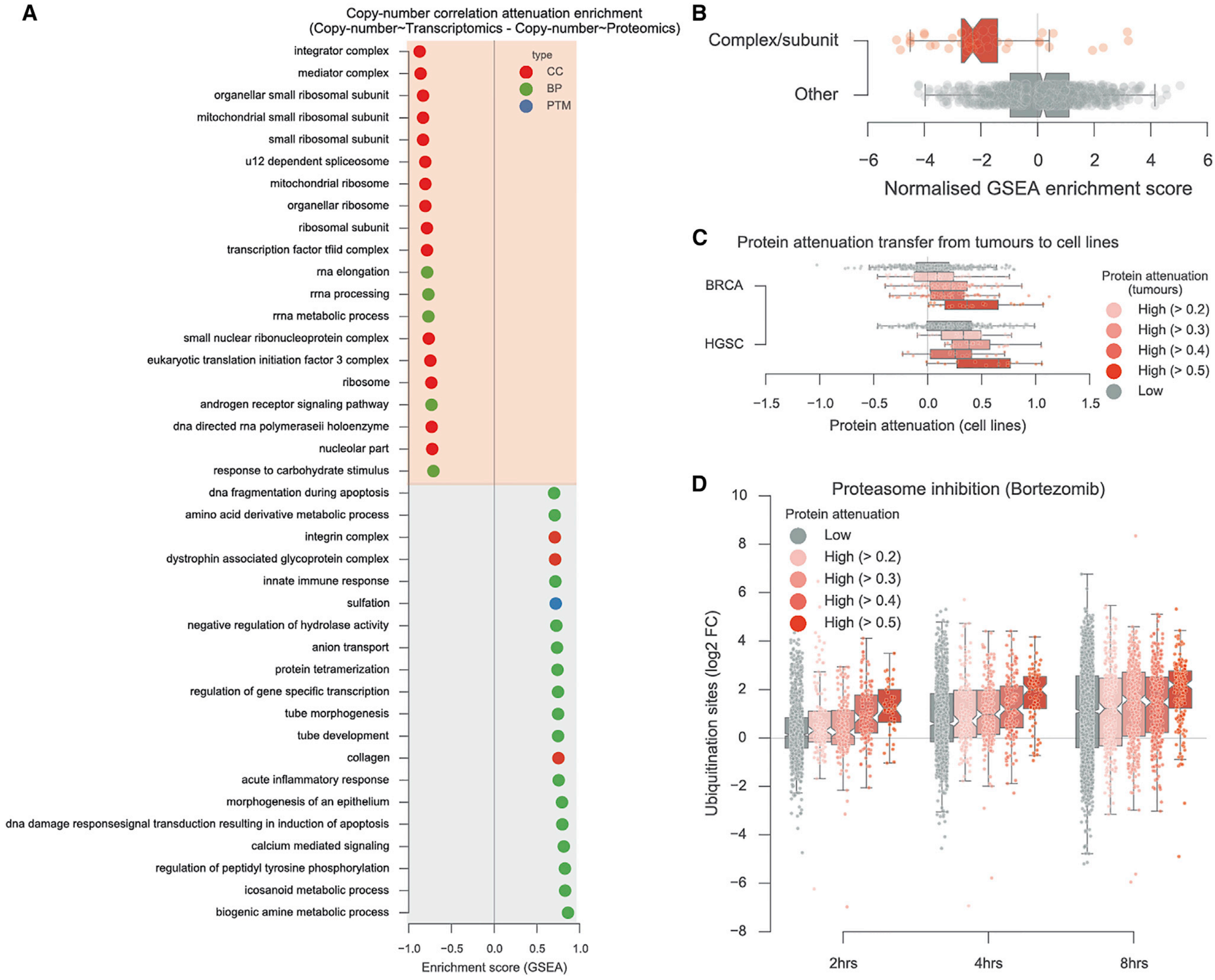

Figure 2. Enrichment Analysis of the Proteins Undergoing Copy-Number Attenuation

(A) Enrichment analysis of the correlation differences between copy-number variation and transcriptomics and copy-number variation and proteomics. Protein subsets used represent biological processes (BP, green), cellular components (CC, red), and post-translational modifications (PTM, blue). Gene sets listed are all significantly enriched at FDR <5%.

(B) The distribution of the enrichment scores for terms referring to protein complexes or subunits are represented in red and all the rest in gray.

(C) Proteins classified according to their attenuation profile in tumors are mapped against their attenuation in breast and ovarian cancer cell lines.

(D) Ubiquitination site fold changes over time after proteasome inhibition with bortezomib discretized according the protein attenuation level in tumors.

pairs that have functional interactions but are not complex subunits show a lower degree of abundance correlation (mean Pearson's r = 0.15) that is also closer to the observed at the transcript level (mean Pearson's r = 0.11) (Figure 3B).

In light of this agreement between functionally related proteins we examined the capacity of protein-protein correlation profiles to predict different types of protein-protein interactions (Figure 3C) (see the STAR Methods). We found that direct and indirect functional interactions could be well identified with proteomics (area under the receiving operating characteristic curves AROC = 0.86 and 0.75, respectively), and worse with transcriptomics (AROC = 0.69 and 0.67, respectively) (Figure 3C). This finding goes in line with a recent work that showed that proteins within similar biological processes or pathways display better agreement at the protein than at the transcript level (Wang et al., 2016). We noticed that protein interactions derived from signaling networks displayed in general poor agreement at the protein and transcript abundance levels (AROC = 0.55 and 0.54) (Figure 3C), suggesting that the abundance of signaling proteins in the same pathway does not necessarily need to be coordinated. Furthermore, metabolic enzymes involved in the same metabolic pathways displayed some degree of agreement at the protein and transcript level (AROC = 0.65 and 0.62) (Figure 3C).

Our results shown that protein complex subunits often have copy-number changes that are attenuated at the protein level and that nevertheless also show higher co-regulation of protein abundance than observed at mRNA level.

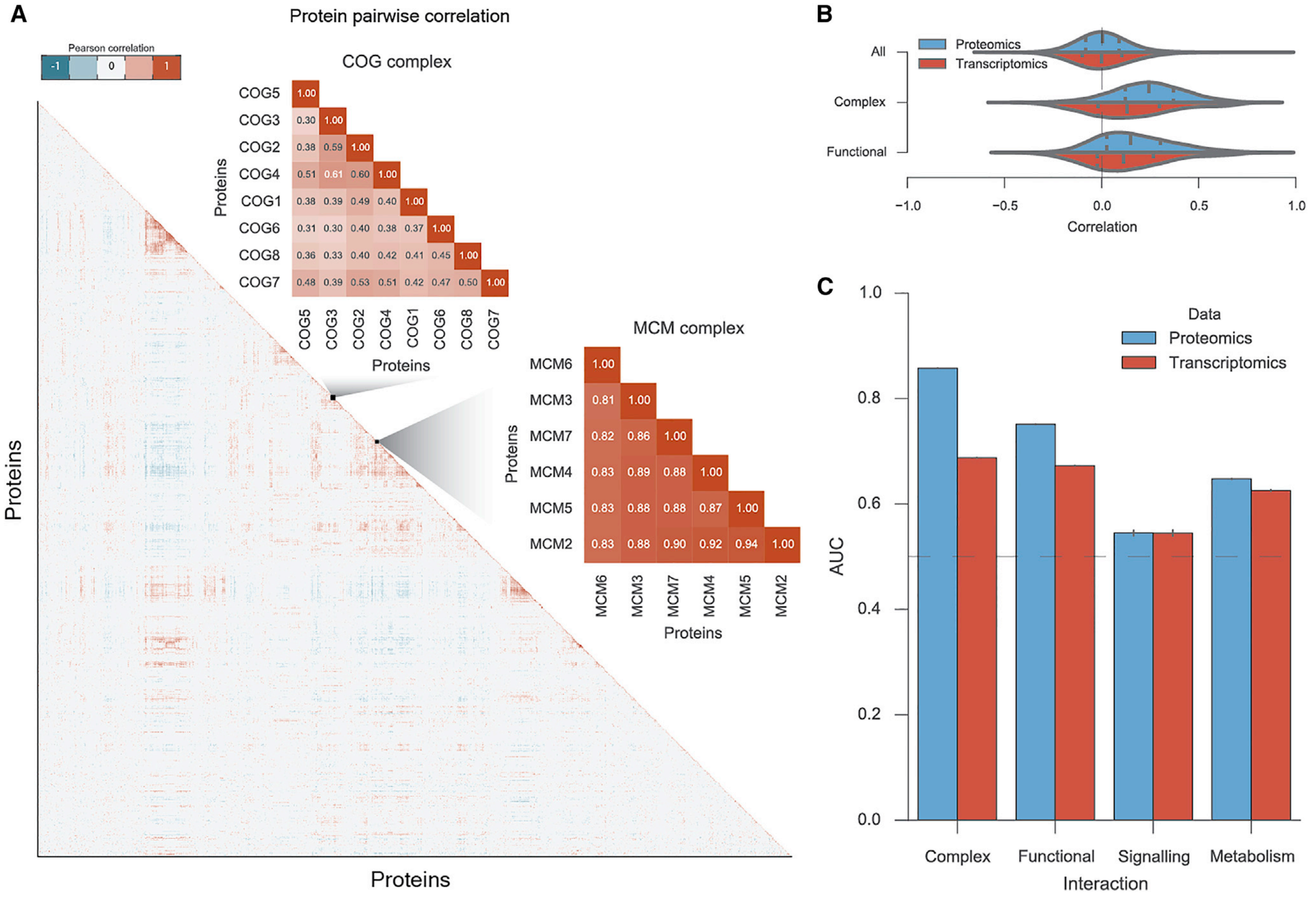

Figure 3. Copy-Number Variation Attenuation for Protein Complex Subunits Results in Strong Co-regulation of Their Abundances across Samples

(A) Protein-protein correlation matrix using Pearson correlation coefficient and two representative cases of top correlated protein complexes.

(B) Distribution of all protein-protein correlations at the protein level (proteomics) and transcript level (transcriptomics). Protein interactions within complexes are represented by the complex label, and protein functional interactions, which are not necessarily direct, are represented by the functional label.

(C) Enrichment analysis by the means of the area under the receiving operating characteristic curves (AROC) using pairwise correlation coefficients, for both proteomics and transcriptomics measurements. Error bars display the variability obtained with five randomized true negative sets.

Proteogenomics Analysis Identifies Subunits that Control the Protein Abundance Levels of Other Members of the Complex

It has been hypothesized that non-attenuated subunits could act as scaffolding proteins or rate-limiting for the assembly of the complex (Dephoure et al., 2014). However, past studies based on aneuploidy models were conducted on a small number of yeast strains or cell lines (Dephoure et al., 2014; Stingele et al., 2012). Given the large number of tumor samples analyzed here we reasoned that we could more readily identify such subunits that can act as drivers of complex assembly. To study this we assessed if the CNVs of a given gene product within a protein complex could explain the changes in abundance of other subunits once we discount their transcriptional changes (see the STAR Methods). In other words, if the presence or absence of certain proteins of the complex could be associated with the protein degradation rate of other members. This was performed systematically for all identifiable protein pairs within protein complexes using linear regression models where the CNVs of a protein (Px) was used to estimate the protein abundance variation of the paired protein (Py) (Figure 4A) (see the STAR Methods). To consider the differences in degradation or translation rates of the protein, the transcript measurements were regressed-out from the protein abundance measurements (Figure 4A) (see the STAR Methods). This allowed us to consider the variability arising post-transcriptionally and, importantly, to discard possible confounding effects occurring at the genomic and transcript level, such as close genomic localization. Out of the 58,627 possible directed protein interactions, 64 were found to be significantly associated (false-discovery rate FDR <5%) (Figure 4A; Table S3) (see the STAR Methods). To ensure that the association was not only visible at the genomic but also at the transcript level, the same associations were performed using transcriptomics measurements. As expected since that transcript abundance is a closer measurement to the protein abundance, we found a substantial increase of significant associations, 2,846 (FDR <5%) (Figure S3; Table S3). Also, 75% (48) of the associations found at the genomic level were found to be significant at the transcript level (Figure 4A; Table S3).

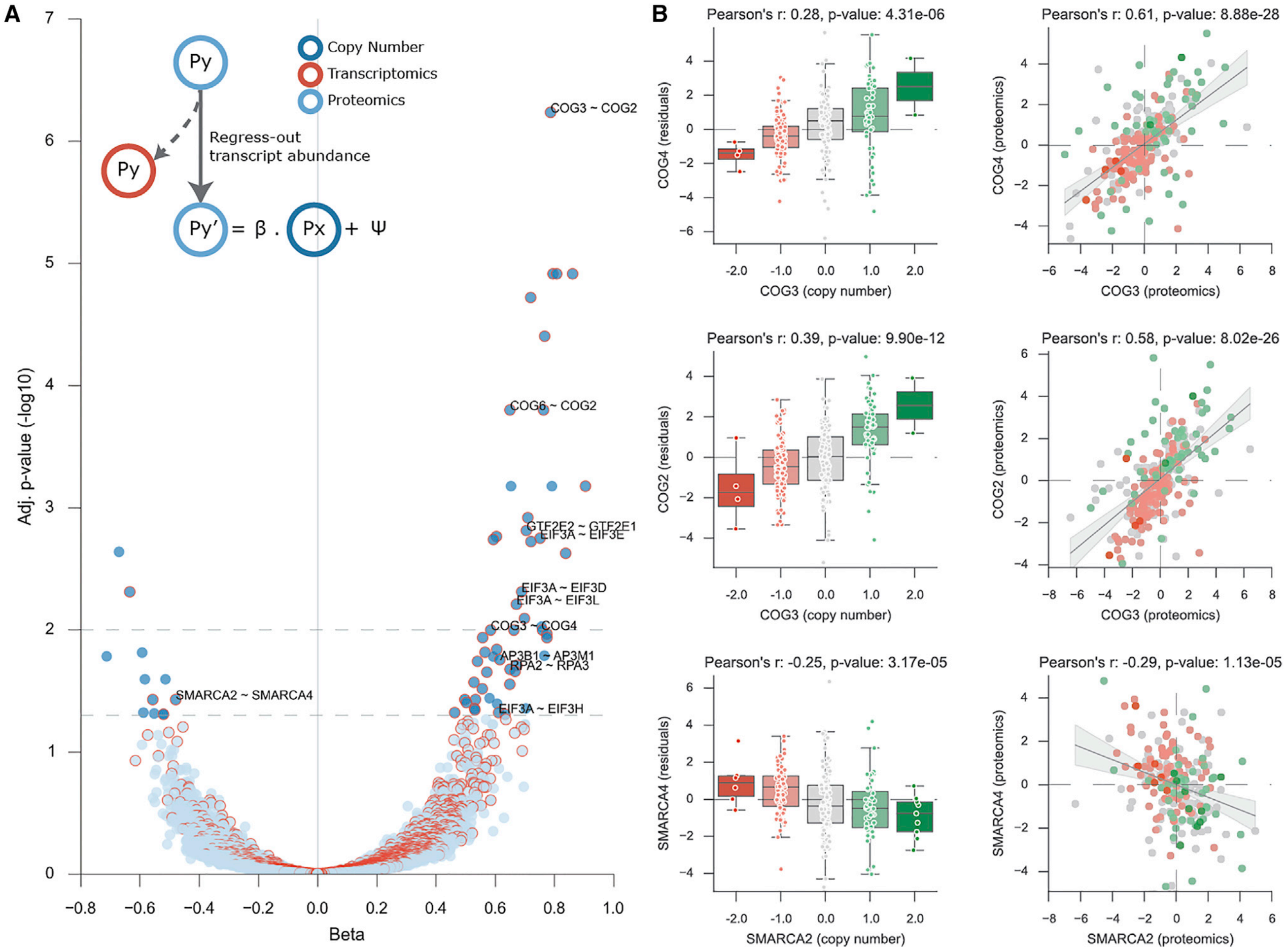

Figure 4. Protein Complex Regulators

(A) Volcano plot displaying the effect size and adjusted p value of all the tested regulatory interactions. Associations were performed using the copy-number variation of the putative regulatory protein, Px, and the protein residuals of the regulated protein, Py. Significant associations found with the transcript measurements of Px are denoted with a red border.

(B) Representative significant associations. Boxplots show the agreement between the copy-number variation of Px and the residuals of the regulated Py. Scatterplot show the agreement between the protein pairs in the proteomics measurements.

Given that the associations are made between the copy level of one gene and the residual abundance of the interactor partner they are expected to be causal relationships. It is unlikely that the residual abundance of a protein would cause a change in the DNA copy number of the interacting partner. Therefore, this analysis identified interactions that may act as rate-limiting steps of the assembly of protein complexes. We found, for example, an association between the copy number of COG3 and the protein variability of COG2 (Pearson's r = 0.39, p value 9.90×10^{12}) (Figure 4B). COG3 is also significantly associated with COG4 (Figure 4B), increasing the possibility that COG3 is a regulator of the assembly of the conserved oligomeric Golgi (COG) complex. These findings are corroborated by an existing study where COG3 knockdown leads to a decreased abundance of COG2 and COG4 (Bailey Blackburn et al., 2016; Zolov and Lupashin, 2005). Besides identifying known rate-limiting members of complexes, our analysis also predicts two possibly novel associations within the COG complex, with COG6 being significantly associated with COG2 (Figure 4). Additional positive regulatory interactions were found for subunits of the eukaryotic initiation factor 3 (EIF3), transcription factor IIH, adaptor-related protein complex 3 (AP3), among others (Table S3), providing with information on the putative assembly pathways of these complexes.

The number of significant negative associations was lower than the number of positive associations (Figures 4A and S3C). SMARCA2 copy-number alterations were significantly and negatively associated with the degradation of SMARCA4 (Figure 4A) and this was also visible at the protein level (Figure 4B). Negative associations are likely to represent mutually exclusive events within protein complexes, thus when one protein is present the other will not be necessary for the complex formation and may undergo degradation. Indeed, current evidence in the literature suggest that SMARCA2 and SMARCA4 are paralogs and mutually exclusive within the SWI/SNF complex (Karnezis et al., 2016; Ori et al., 2016). The lower number of negative associations suggests that these types of events are less frequent.

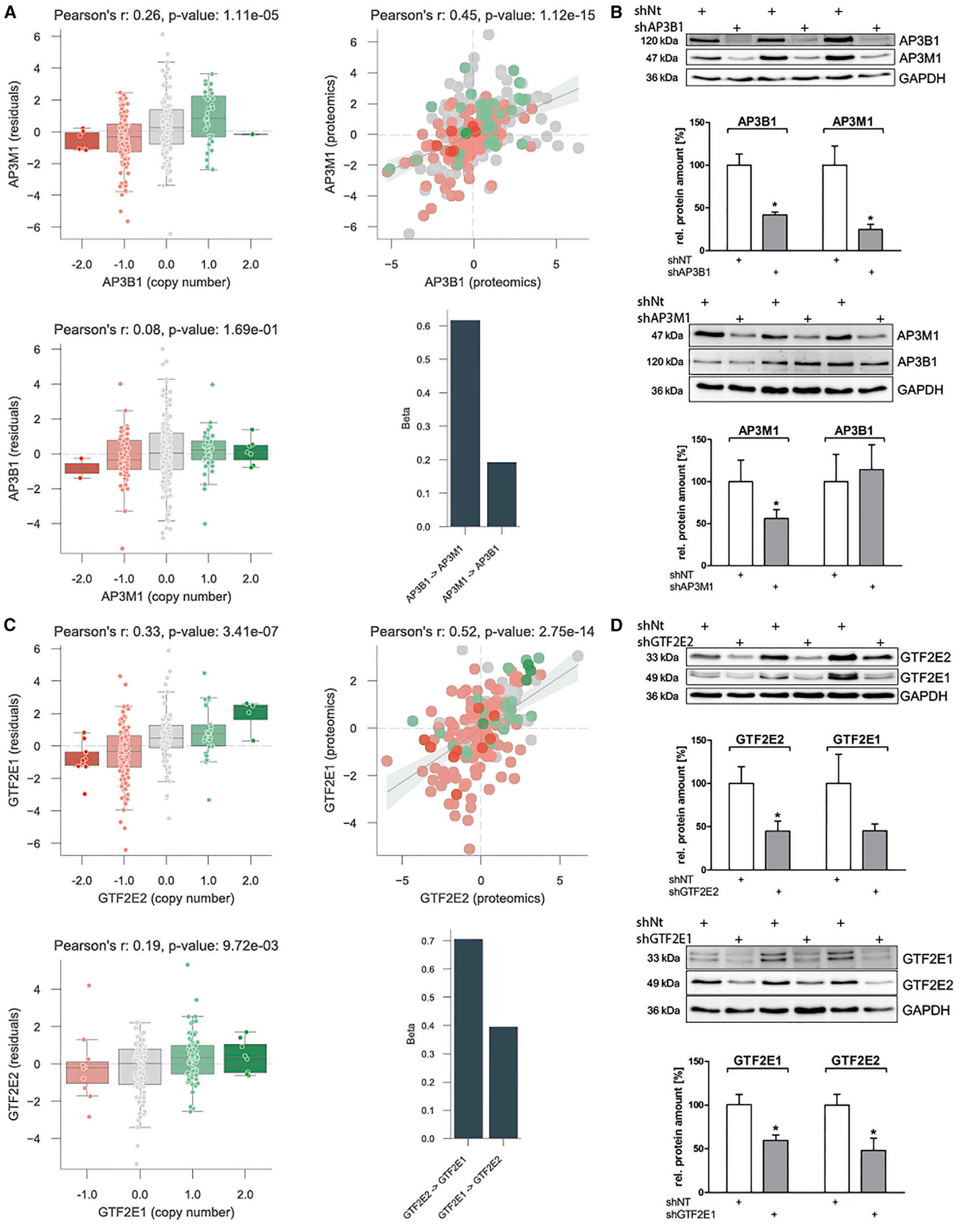

(legend on next page)

AP3B1 and GTF2E2 Protein Abundance Levels Indirectly Control the Abundance of Interaction Partners

We experimentally validated two of the top significant positive associations (Figure 5). These were found within protein complex subunits of the AP3 and the transcription initiation factor IIE (TFIIE), AP3B1-AP3M1, and GTF2E2-GTF2E1, respectively (Figures 5A and 5C). To assess their implication we performed small hairpin RNA (shRNA) knockdown of the putative rate-limiting proteins, AP3B1 and GTF2E2, in shRNA transfected HCT116 human colon cancer cell lines followed by western blot. Knocking down AP3B1 and GTF2E2 not only affected their abundance but also the abundance of the interacting proteins within the protein complex subunit, AP3M1 and GTF2E1 (Figures 5B and 5D). While for the putative rate-limiting interactions the inverse association was not found significant (FDR >5%), we cannot exclude that they might exist as we are limited by the coverage of the datasets. For example, the lack of variability at the copy-number level might lead to uninformative associations of the gene product with the other members of the complex. To address this, we also performed the reverse experiment by knocking down AP3M1 and GTF2E1 and measured the impact in protein abundance. We observed that AP3M1 knockdown did not have any impact in the abundance of AP3B1 (Figure 5B) as expected by the low association coefficient of the linear model (Figure 5A). On the other hand, GTF2E1 knockdown resulted in the depletion of GTF2E2 (Figure 5D) suggesting that this rate-limiting interaction is bidirectional. The lack of any strong depletion of GTF2E1 in the copy-number dataset may explain why this association cannot be captured on this direction (Figure 5C).

To further assess if our associations were capable of identifying the rate-limiting interactions occurring in both directions we used two independent studies where members of COG and EIF3 were systematically knocked down with shRNAs, and the abundance of the complex members was measured with western blot (Bailey Blackburn et al., 2016; Wagner et al., 2014). We found a significant (Spearman's $r = -0.4$, p value 3.4×10^{-4}) agreement between our predicted association effect and those measured experimentally (Figure 6A). Moreover, all the significant associations captured within these complexes showed significantly higher impact on abundance (Figure 6B). This highlighted that our approach is able to capture well rate-limiting associations with strong effects and can identify with moderate confidence if the association occurs in both directions.

Molecular Features Associated with High Attenuation Potential

Having assessed the attenuation of the effects of CNVs in the proteome we set out to quantify the extent of this regulation in each tumor sample. We reasoned that, by stratifying the samples by their capacity to attenuate the CNV changes, we could identify the underlying attenuation mechanisms. Similarly to the protein analysis (Figure 1D), we performed a correlation analysis between the CNVs and transcriptomics and proteomics for each sample (Figure 7A), instead of each protein. Furthermore, recurring to a Gaussian mixture model we classified 50 samples (18%) as those having a general strong attenuation effect (see the STAR Methods). Such tumor samples have a higher number of genes with strong attenuation, suggesting either an overall increase in degradation or decrease in translation rates in these samples. To attempt to understand the underlying differences in attenuation potential we first correlated this metric with the degree of somatic copy-number alterations from Davoli et al. (2017) and observed a significant correlation ($r = 0.33$, p value = 1.2×10^{-7}). This would suggest that in part the higher apparent attenuation potential is due to larger copy-number alterations. It also indirectly suggests that there is not a very strong saturation whereby larger numbers of gene-dosage alterations would result in lower attenuation capacity. We did not find a significant association between attenuation potential and sample ploidy or sample purity ($r = 0.031$ and -0.11, respectively, Figures S4B and S4C). We then searched for complexes and complex subunits that are more likely to be amplified or deleted in the tumors with stronger attenuation and could therefore contribute to the attenuation potential (see the STAR Methods). Tumors with strong attenuation effects displayed a significant enrichment of gene amplifications in several complex subunits, including genes involved in the endoplasmic reticulum-associated degradation (ERAD) pathway (DERL1 and VIMP), cell polarity (SCRIB, LLGL2, and VANGL2), GPI-anchor biosynthesis (PIGT and PIGU), and RNAi (AGO2) (Figures 7B and 7C). We also found significant enrichment for deletions in GTF2E2 involved in transcription regulation complex TFIIE.

Gene Expression Profile of Protein Attenuation Is Associated with Specific Drug Responses

Since the tumors with strong attenuation of the effects of CNVs displayed particular characteristics, we defined a gene expression signature by systematically correlating each gene with the attenuation potential (see the STAR Methods). We then performed gene set enrichment analysis on this gene expression signature (Figures S4E and S4F) and we found that samples with higher attenuation potential have increased expressions of cell-cycle-related functions (e.g., meiotic recombination, sister chromatid segregation, G1 phase of the mitotic cell cycle), and decreased expression of metabolic-related function (e.g., phagocytosis, respiratory chain complex I, and glucosamine

Figure 5. Experimental Validation of Regulatory Interactions among Protein Complex Subunits

Rate-limiting interactions within the adaptor protein complex 3 (AP3) and the transcription initiation factor IIE (TFIIE) complexes.

(A and C) Correlation of the copy-number profile of the regulatory protein with the protein residuals of the regulated protein (left plot) and agreement at the protein level between the two proteins (right plot).

(B and D) shRNA knockdown of the regulatory proteins, AP3B1 and GTF2E2, show strong decrease in the protein abundance of the regulated proteins, AP3M1 and GTF2E1, respectively. Knocking down GTF2E1 showed a significant downregulation of GTF2E2, indicating a bidirectional relation between those proteins. In contrast, AP3M1 shRNA did not affect AP3B1 protein abundance. Protein abundance changes are measured and quantified by western blot using antibodies specific for the corresponding proteins. The quantified bands in the shAP3B1, shAP3M1, shGTF2E2, and shGTF2E1 experiments were scored relative to the control shRNA (shNT). GAPDH was used as a loading control.

Error bars shown are the SD from the mean ($n = 3$ independent experiments). *$p < 0.05$ compared with shNT, two-tailed unpaired t test.

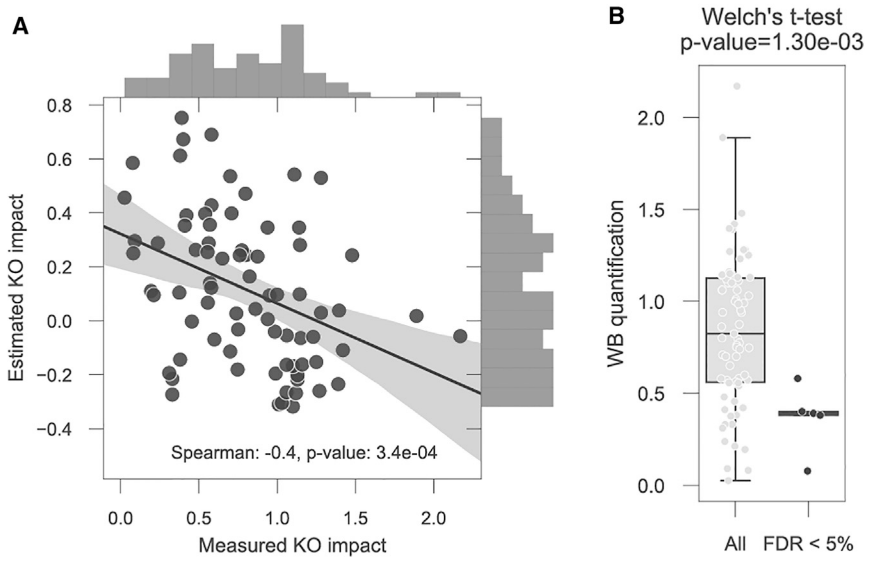

Figure 6. COG3 and EIF3 Complexes Rate-Limiting Interactions

(A) Agreement between experimentally measured COG and EIF3 complex element knockdown with *in silico* estimated impact.

(B) Welch's t test comparing the computational rate-limiting interactions (FDR <5%) and all the other experimentally measured interactions.

metabolic process). Among the downregulated functions are also some related to immune response (e.g., cytokine secretion and cellular defense response). This is consistent with the observation that samples with higher somatic copy-number alterations have downregulation of immune-related gene sets (Davoli et al., 2017). However, while our measure of attenuation potential per sample is correlated with total SCNAs scores, it is not correlated with sample purity (r = −0.11, p value = 8.5 × 10^{-2}), indicating that there is no strong difference of immune infiltration across samples of different attenuation potential. These changes in gene expression are more likely reflective of the degree of copy-number alterations and may not be immediately informative to understand the mechanisms underlying the differences in attenuation potential. We observed that this signature is capable of discriminating samples with strong versus weak attenuation using a cross-validation approach (Figure S4A; AROC = 0.69). This signature provides a putative ranking of the agreement between gene expression and the attenuation profile of the samples. Next, we explored the capacity of this signature to identify particular cellular states that can be informative for drug response. Samples with a strong correlation with the signature would be predicted to have higher attenuation and could, for example, display a higher proteasomal capacity. Thus, we considered an independent cell line panel for which gene expression and drug response is available (Iorio et al., 2016b), and ranked the cell lines according to their predicted protein attenuation potential (see the STAR Methods). Then we assessed the association between this predicted attenuation potential and drug-response measurements for 265 compounds (see the STAR Methods) (Figures 7D and S4D). Among the top predicted compounds are a proteasome (Bortezomib and MG-132) and chaperone inhibitors (AUY922, 17-AAG, Elesclomol, CCT018159, and SNX-2112), which displayed a significant (FDR <5%) positive association, suggesting that a stronger predicted attenuation potential is associated with increased resistance to proteasome/chaperone inhibitors (Table S4). This unbiased search also revealed significantly positive associations of Nutlin-3a and JNJ-26854165 and the proteome attenuation profile (Pearson's r = 0.20 and 0.16, respectively). Both compounds target the oncoprotein E3 ligase MDM2 which, in p53 wild-type tumors, suppresses the activity of p53 by ubiquitination and thereby is a potential therapeutic target (Shangary and Wang, 2008). The protein attenuation potential predicted for the cell lines also displayed tissue specificity, supporting the idea that proteasomal capacity is constrained by the tissue of origin. This analysis suggests that the gene expression signature for the proteome attenuation may be associated with an increased capacity of the protein quality control machinery and an increased resistance to drugs that target this system.

DISCUSSION

Gene-Dosage Changes Are Attenuated for 23%–33% of Proteins

We aimed here to study the extent by which gene dosage is attenuated in cancer at the protein level and what are the mechanisms that govern this process. We observed that, while CNVs have on average a good agreement with transcript measurements, 23%–33% of the proteins undergo post-transcriptional regulation, which attenuates the impact of CNVs (Figures 1C and 1D). We cannot rule out the possibility that some of the apparent protein level attenuation may be due to higher measurement error in the protein abundance relative to the gene expression measurements. However, this is not expected to alter the ranking of proteins from strongest to weakest attenuation as shown by the replication with the cell line data (Figure 2C). The identification of attenuated proteins alone is very relevant for the identification of causal genes within amplified genome regions. Since copy-number changes are buffered and not observed at the protein level, these are therefore less likely to be drivers of cancer progression and similarly less likely to explain changes in drug associations. Notably, this attenuation was more pronounced in protein subunits and complexes, in agreement with previous observations (Dephoure et al., 2014; Stingele et al., 2012). This is likely explained by the fact that the stoichiometry of complexes needs to be preserved, and that proteins over-represented compared with other members of the complex are likely degraded due to increased instability (McShane et al., 2016). Furthermore, we observed that proteins with stronger attenuation are more quickly ubiquitinated (Kim et al., 2011) (Figure 2D), suggesting that the attenuation may be mostly driven by changes in degradation instead of translation rates. In line with this, it has been shown, in time-series

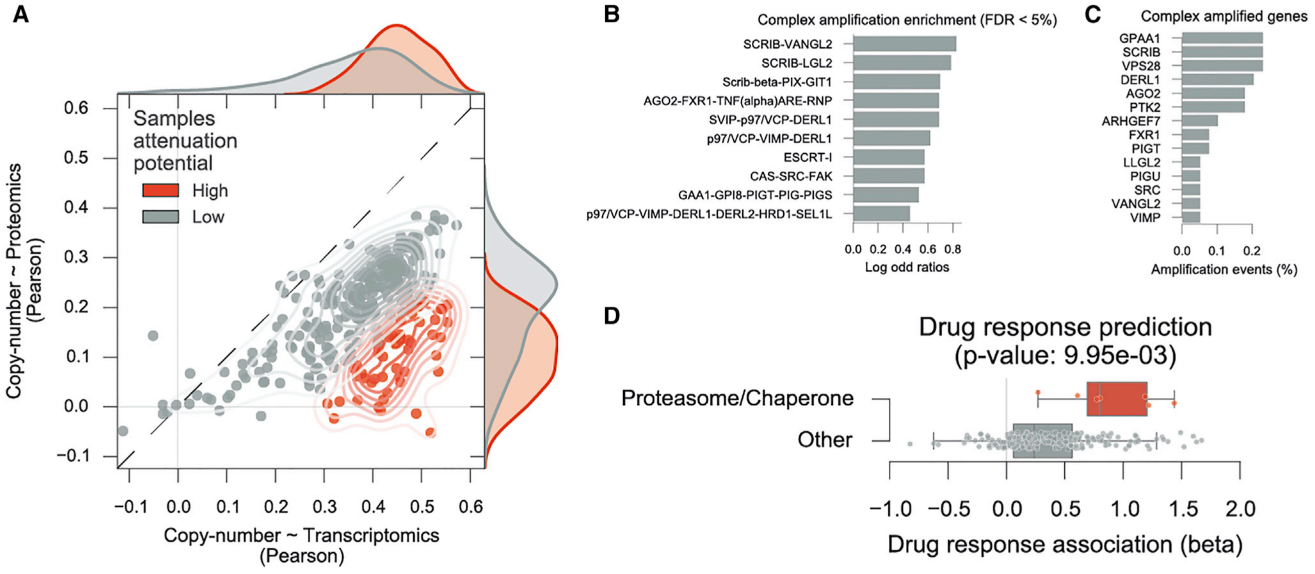

Figure 7. Putative Mechanisms for Tumor Attenuation Potential and Their Association with Chaperone/Proteasome Drug Resistance

(A) Tumor sample correlations of the copy-number changes and the transcript (x axis) and protein (y axis) measurements. Samples classified with high attenuation potential, in red, display stronger attenuation of the copy-number variation.

(B) Protein complexes significantly enriched for gene amplifications (FDR <5%) on the samples with high protein attenuation.

(C) Top strongly amplified genes within the significantly enriched complexes.

(D) Drug-response associations performed in a large cell line panel using the cell lines using putative attenuation potential as the predictive feature. Significant associations (FDR <5%) of chaperone and proteasome inhibitors are labeled and marked in red. Boxplots representing the distributions of the drug associations effect sizes of all the proteasome and chaperones inhibitors in the drug panel.

experiments, that many protein complex subunits have degradation profiles that are best fit by a two-state model, suggesting that the degradation rate of these proteins changes, presumably when free or when assembled into the complex (McShane et al., 2016). Attenuation of abnormal gene copy numbers by protein degradation seems to be a general and conserved effect in aneuploidy cells, as also shown in McShane et al. (2016). We note that we cannot rule out that control of translation rate might also play an important role to buffer copy-number alterations.

Some Proteins Can Indirectly Control the Abundance of Interaction Partners

We identified 48 putative rate-limiting proteins for complex assembly, capable of regulating the abundance of other complex subunits (Figure 4A). These results suggest that protein interactions and complex assembly are important control points for protein level gene-dosage compensation. This systematic analysis recapitulated previously known rate-limiting interactions in COG and EIF3, and it also found potentially novel associations. Of these, we have experimentally validated two rate-limiting interactions, AP3B1-AP3M1 and GTF2E2-GTF2E1, within the AP3 and TFIIE complexes, respectively (Figure 5). The AP3B1-AP3M1 interaction was not bidirectional in contrast to the GTF2E2-GTF2E1. This latter case is of particular importance as it illustrates a case where we did not predict but observed an indirect effect on abundance of an interacting protein. The absence of a predicted indirect effect could be due to lack of statistical power, for example a limited number of strong depletions and amplifications of a given gene. We also designed experimental validations for RPA2-RPA3 and for EIF3A-EIF3E, but knocking down RPA2 or EIF3A proved to be lethal for the transfected HCT116 colon cancer cell lines. Potential mutual exclusivity associations were present in much lower numbers. The most compelling negative association was SMARCA2-SMARCA4, which was supported by current literature where the two are reported to be mutually exclusive ATPases (Karnezis et al., 2016) and paralogs (Ori et al., 2016) within the SWI/SNF complex.

Identification of *trans*-regulatory effects is still a challenging task and it is estimated to represent 70% of mRNA heritability (Price et al., 2011). These results provide examples and putative mechanistic explanations for how variation in copy number or gene expression of a protein can have *trans* effects in the abundance of interacting proteins, as seen in protein quantitative trait loci analyses (Battle et al., 2015; Chick et al., 2016). Identification of rate-limiting interactions in protein complex assembly will help understand how protein-protein interactions are structured and will be important to understand complex traits (Boyle et al., 2017).

Association Analysis Suggests Mechanisms Associated with Gene-Dosage Attenuation

Tumor samples with strong attenuation of the effects of CNVs in protein abundance displayed a significant enrichment for amplifications of several protein complexes involved in the response to misfolded proteins in the endoplasmic reticulum (ER), cell polarity, trafficking, and gene repression. Consistent with the increased protein attenuation profile of these tumors, we observe amplifications of the ERAD components, DERL1 and VIMP, which are part of an ER complex that is responsible for the retrotranslocation of misfolded proteins to the cytosol for

proteasomal degradation (Lilley and Ploegh, 2004; Ye et al., 2004). While this association is expected, the others are less obviously linked to post-transcriptional control. The cell polarity-related SCRIB protein complexes have been previously reported to play an important role in cancer progression in breast cancer, and their inhibition has been linked to a decrease in cell migration (Anastas et al., 2012). The proteasome system is important for the regulation of focal adhesions in migrating cells (Teckchandani and Cooper, 2016), and inhibition of the proteasome inhibits migration and invasion in breast cancer cells (Xie et al., 2009). However, it is not clear how the overexpression of these cell polarity factors would result in an increase in attenuation potential. The association between increased attenuation and amplification of AGO2 could be explained by its role in repressing the initiation of mRNA translation (Kiriakidou et al., 2007).

Differential Drug-Response Association with Gene Expression Signature of Proteome Attenuation

In cell lines, proteome attenuation, predicted by a gene expression signature, was associated with increased resistance to proteasome and chaperone inhibitors (Figure 7D), suggesting that tumors, where attenuation is more pronounced, are more resistant to perturbations in the chaperone/proteasome system. The two compounds in the screen targeting MDM2 were among the top associated with the gene expression signature, suggesting that tumors with high predicted attenuation potential may have a high proteasome capacity and therefore be less sensitive to the inhibition of MDM2, which is the E3 ligase responsible for the degradation of TP53 in p53 wild-type tumors (Shangary and Wang, 2008). While we show that the gene expression signature has some power to predict attenuation potential in cross-validation tests, additional work will be required to conclusively validate the putative associations between the attenuation potential and the drug responses. The increasing availability of proteomics studies in cancer cell lines will enable the estimation of protein attenuation directly and without the need to rely on an attenuation potential gene expression signature defined in tumor samples. This will augment our power to study gene-dosage compensation and its effect on drug response.

In this study, we provide insights into how cancer cells manage to cope with often dramatic chromosomal rearrangements (Thompson and Compton, 2011), and these can possibly provide insights into their functional implications and hopefully open novel therapeutic opportunities.

STAR★METHODS

Detailed methods are provided in the online version of this paper and include the following:

- KEY RESOURCES TABLE
- CONTACT FOR REAGENT AND RESOURCE SHARING
- EXPERIMENTAL MODEL AND SUBJECT DETAILS
- METHOD DETAILS
  - Cell Lines Drug Response Analysis
  - shRNA Delivery via Lentiviral Transduction
  - Western Blot Validation
- QUANTIFICATION AND STATISTICAL ANALYSIS
  - Data Compendium
  - Data Processing and Normalisation
  - Proteome Attenuation Analysis
  - Pairwise Correlation Analysis
  - Proteogenomics Analysis to Identify Protein Complex Regulators
  - Logistic Classification of Samples Protein Attenuation Potential
  - Statistical Analysis of Experimental Data
  - Code Availability

SUPPLEMENTAL INFORMATION

Supplemental Information includes four figures and four tables and can be found with this article online at https://doi.org/10.1016/j.cels.2017.08.013.

AUTHOR CONTRIBUTIONS

J.S.R. and P.B. conceived and led the study. E.G. carried out the analysis. A.F. and T.C. designed the experimental validations. A.F. carried out cell cultures and knocking down experiments. L.G.A. contributed to the analysis. E.G., J.S.R., and P.B. wrote the paper.

ACKNOWLEDGMENTS

We thank Michael Schubert for help integrating the copy-number variation data and Paolo Casale for helping define the linear models. We gratefully acknowledge helpful comments from Colm Ryan, Marc Brehme, David Ochoa, Danish Memon, Romain Studer, Haruna Imamura, and Theodoros Roumeliotis. We thank Jessica Bailey and Vladimir Lupashin for kindly providing experimental measurements on the COG knockdown experiment.

Received: February 1, 2017
Revised: June 21, 2017
Accepted: August 23, 2017
Published: October 11, 2017

REFERENCES

Abovich, N., Gritz, L., Tung, L., and Rosbash, M. (1985). Effect of RP51 gene dosage alterations on ribosome synthesis in *Saccharomyces cerevisiae*. Mol. Cell. Biol. *5*, 3429–3435.

Anastas, J.N., Biechele, T.L., Robitaille, M., Muster, J., Allison, K.H., Angers, S., and Moon, R.T. (2012). A protein complex of SCRIB, NOS1AP and VANGL1 regulates cell polarity and migration, and is associated with breast cancer progression. Oncogene *31*, 3696–3708.

Ashburner, M., Ball, C.A., Blake, J.A., Botstein, D., Butler, H., Michael Cherry, J., Davis, A.P., Dolinski, K., Dwight, S.S., Eppig, J.T., et al. (2000). Gene ontology: tool for the unification of biology. Nat. Genet. *25*, 25–29.

Avner, P., and Heard, E. (2001). X-Chromosome inactivation: counting, choice and initiation. Nat. Rev. Genet. *2*, 59–67.

Bailey Blackburn, J., Pokrovskaya, I., Fisher, P., Ungar, D., and Lupashin, V.V. (2016). COG complex complexities: detailed characterization of a complete set of HEK293T cells lacking individual COG subunits. Front. Cell Dev. Biol. *4*, 23.

Battle, A., Khan, Z., Wang, S.H., Mitrano, A., Ford, M.J., Pritchard, J.K., and Gilad, Y. (2015). Genomic variation. Impact of regulatory variation from RNA to protein. Science *347*, 664–667.

Beroukhim, R., Mermel, C.H., Porter, D., Wei, G., Raychaudhuri, S., Donovan, J., Barretina, J., Boehm, J.S., Dobson, J., Urashima, M., et al. (2010). The landscape of somatic copy-number alteration across human cancers. Nature *463*, 899–905.

Boyle, E.A., Li, Y.I., and Pritchard, J.K. (2017). An expanded view of complex traits: from polygenic to omnigenic. Cell *169*, 1177–1186.

Cancer Genome Atlas Network. (2012a). Comprehensive molecular characterization of human colon and rectal cancer. Nature *487*, 330–337.

Cancer Genome Atlas Network. (2012b). Comprehensive molecular portraits of human breast tumours. Nature *490*, 61–70.

Cancer Genome Atlas Research Network. (2011). Integrated genomic analyses of ovarian carcinoma. Nature *474*, 609–615.

Chick, J.M., Munger, S.C., Simecek, P., Huttlin, E.L., Choi, K., Gatti, D.M., Raghupathy, N., Svenson, K.L., Churchill, G.A., and Gygi, S.P. (2016). Defining the consequences of genetic variation on a proteome-wide scale. Nature *534*, 500–505.

Coscia, F., Watters, K.M., Curtis, M., Eckert, M.A., Chiang, C.Y., Tyanova, S., Montag, A., Lastra, R.R., Lengyel, E., and Mann, M. (2016). Integrative proteomic profiling of ovarian cancer cell lines reveals precursor cell associated proteins and functional status. Nat. Commun. *7*, 12645.

Davoli, T., Uno, H., Wooten, E.C., and Elledge, S.J. (2017). Tumor aneuploidy correlates with markers of immune evasion and with reduced response to immunotherapy. Science *355*, https://doi.org/10.1126/science.aaf8399.

Dephoure, N., Hwang, S., O'Sullivan, C., Dodgson, S.E., Gygi, S.P., Amon, A., and Torres, E.M. (2014). Quantitative proteomic analysis reveals posttranslational responses to aneuploidy in yeast. Elife *3*, e03023.

Edwards, N.J., Oberti, M., Thangudu, R.R., Cai, S., McGarvey, P.B., Jacob, S., Madhavan, S., and Ketchum, K.A. (2015). The CPTAC data portal: a resource for cancer proteomics research. J. Proteome Res. *14*, 2707–2713.

Franceschini, A., Szklarczyk, D., Frankild, S., Kuhn, M., Simonovic, M., Roth, A., Lin, J., Minguez, P., Bork, P., von Mering, C., et al. (2013). STRING v9.1: protein-protein interaction networks, with increased coverage and integration. Nucleic Acids Res. *41*, D808–D815.

Hunter, J.D. (2007). Matplotlib: a 2D graphics environment. Comput. Sci. Eng. *9*, 90–95.

Iorio, F., Knijnenburg, T.A., Vis, D.J., Bignell, G.R., Menden, M.P., Schubert, M., Aben, N., Gonçalves, E., Barthorpe, S., Lightfoot, H., et al. (2016a). A landscape of pharmacogenomic interactions in cancer. Cell *166*, 740–754.

Iorio, F., Garcia-Alonso, L., Brammeld, J., Martincorena, I., Wille, D.R., McDermott, U., and Saez-Rodriguez, J. (2016b). Pathway-based dissection of the genomic heterogeneity of cancer hallmarks with SLAPenrich. BioRxiv. https://doi.org/10.1101/077701.

Itsara, A., Cooper, G.M., Baker, C., Girirajan, S., Li, J., Absher, D., Krauss, R.M., Myers, R.M., Ridker, P.M., Chasman, D.I., et al. (2009). Population analysis of large copy number variants and hotspots of human genetic disease. Am. J. Hum. Genet. *84*, 148–161.

Kanehisa, M., Sato, Y., Kawashima, M., Furumichi, M., and Tanabe, M. (2016). KEGG as a reference resource for gene and protein annotation. Nucleic Acids Res. *44*, D457–D462.

Karnezis, A.N., Wang, Y., Ramos, P., Hendricks, W.P., Oliva, E., D'Angelo, E., Prat, J., Nucci, M.R., Nielsen, T.O., Chow, C., et al. (2016). Dual loss of the SWI/SNF complex ATPases SMARCA4/BRG1 and SMARCA2/BRM is highly sensitive and specific for small cell carcinoma of the ovary, hypercalcaemic type. J. Pathol. *238*, 389–400.

Kim, W., Bennett, E.J., Huttlin, E.L., Guo, A., Li, J., Possemato, A., Sowa, M.E., Rad, R., Rush, J., Comb, M.J., et al. (2011). Systematic and quantitative assessment of the ubiquitin-modified proteome. Mol. Cell *44*, 325–340.

Kiriakidou, M., Tan, G.S., Lamprinaki, S., De Planell-Saguer, M., Nelson, P.T., and Mourelatos, Z. (2007). An mRNA m7G cap binding-like motif within human Ago2 represses translation. Cell *129*, 1141–1151.

Law, C.W., Chen, Y., Shi, W., and Smyth, G.K. (2014). voom: precision weights unlock linear model analysis tools for RNA-seq read counts. Genome Biol. *15*, R29.

Lawrence, R.T., Perez, E.M., Hernández, D., Miller, C.P., Haas, K.M., Irie, H.Y., Lee, S.-I., Blau, C.A., and Villén, J. (2015). The proteomic landscape of triple-negative breast cancer. Cell Rep. *11*, 630–644.

Lilley, B.N., and Ploegh, H.L. (2004). A membrane protein required for dislocation of misfolded proteins from the ER. Nature *429*, 834–840.

Lyon, M.F. (1961). Gene action in the X-chromosome of the mouse (*Mus musculus* L.). Nature *190*, 372–373.

McKinney, W., (2010). Data structures for statistical computing in python. In Proceedings of the 9th Python in Science Conference, pp. 51–56.

McShane, E., Sin, C., Zauber, H., Wells, J.N., Donnelly, N., Wang, X., Hou, J., Chen, W., Storchova, Z., Marsh, J.A., et al. (2016). Kinetic analysis of protein stability reveals age-dependent degradation. Cell *167*, 803–815.e21.

Mermel, C.H., Schumacher, S.E., Hill, B., Meyerson, M.L., Beroukhim, R., and Getz, G. (2011). GISTIC2.0 facilitates sensitive and confident localization of the targets of focal somatic copy-number alteration in human cancers. Genome Biol. *12*, R41.

Mertins, P., Mani, D.R., Ruggles, K.V., Gillette, M.A., Clauser, K.R., Wang, P., Wang, X., Qiao, J.W., Cao, S., Petralia, F., et al. (2016). Proteogenomics connects somatic mutations to signalling in breast cancer. Nature *534*, 55–62.

Nagaraj, N., Wisniewski, J.R., Geiger, T., Cox, J., Kircher, M., Kelso, J., Pääbo, S., and Mann, M. (2011). Deep proteome and transcriptome mapping of a human cancer cell line. Mol. Syst. Biol. *7*, 548.

Ori, A., Iskar, M., Buczak, K., Kastritis, P., Parca, L., Andrés-Pons, A., Singer, S., Bork, P., and Beck, M. (2016). Spatiotemporal variation of mammalian protein complex stoichiometries. Genome Biol. *17*, 47.

Pedregosa, F., Varoquaux, G., Gramfort, A., Michel, V., Thirion, B., Grisel, O., Blondel, M., Prettenhofer, P., Weiss, R., Dubourg, V., et al. (2011). Scikit-learn: machine learning in python. J. Mach. Learn. Res. *12*, 2825–2830.

Perfetto, L., Briganti, L., Calderone, A., Perpetuini, A.C., Iannuccelli, M., Langone, F., Licata, L., Marinkovic, M., Mattioni, A., Pavlidou, T., et al. (2016). SIGNOR: a database of causal relationships between biological entities. Nucleic Acids Res. *44*, D548–D554.

Price, A.L., Helgason, A., Thorleifsson, G., McCarroll, S.A., Kong, A., and Stefansson, K. (2011). Single-tissue and cross-tissue heritability of gene expression via identity-by-descent in related or unrelated individuals. PLoS Genet. *7*, e1001317.

Rahman, M., Jackson, L.K., Johnson, W.E., Li, D.Y., Bild, A.H., and Piccolo, S.R. (2015). Alternative preprocessing of RNA-sequencing data in The Cancer Genome Atlas leads to improved analysis results. Bioinformatics *31*, 3666–3672.

Ritchie, M.E., Phipson, B., Wu, D., Hu, Y., Law, C.W., Shi, W., and Smyth, G.K. (2015). limma powers differential expression analyses for RNA-sequencing and microarray studies. Nucleic Acids Res. *43*, e47.

Robinson, M.D., and Oshlack, A. (2010). A scaling normalization method for differential expression analysis of RNA-seq data. Genome Biol. *11*, R25.

Robinson, M.D., McCarthy, D.J., and Smyth, G.K. (2010). edgeR: a Bioconductor package for differential expression analysis of digital gene expression data. Bioinformatics *26*, 139–140.

Ruepp, A., Brauner, B., Dunger-Kaltenbach, I., Frishman, G., Montrone, C., Stransky, M., Waegele, B., Schmidt, T., Doudieu, O.N., Stümpflen, V., et al. (2008). CORUM: the comprehensive resource of mammalian protein complexes. Nucleic Acids Res. *36*, D646–D650.

Ruepp, A., Waegele, B., Lechner, M., Brauner, B., Dunger-Kaltenbach, I., Fobo, G., Frishman, G., Montrone, C., and Mewes, H.-W. (2010). CORUM: the comprehensive resource of mammalian protein complexes – 2009. Nucleic Acids Res. *38*, D497–D501.

Santarius, T., Shipley, J., Brewer, D., Stratton, M.R., and Cooper, C.S. (2010). A census of amplified and overexpressed human cancer genes. Nat. Rev. Cancer *10*, 59–64.

Shangary, S., and Wang, S. (2008). Targeting the MDM2-p53 interaction for cancer therapy. Clin. Cancer Res. *14*, 5318–5324.

Stingele, S., Stoehr, G., Peplowska, K., Cox, J., Mann, M., and Storchova, Z. (2012). Global analysis of genome, transcriptome and proteome reveals the response to aneuploidy in human cells. Mol. Syst. Biol. *8*, 608.

Subramanian, A., Tamayo, P., Mootha, V.K., Mukherjee, S., Ebert, B.L., Gillette, M.A., Paulovich, A., Pomeroy, S.L., Golub, T.R., Lander, E.S., et al. (2005). Gene set enrichment analysis: a knowledge-based approach for interpreting genome-wide expression profiles. Proc. Natl. Acad. Sci. USA *102*, 15545–15550.

Tang, Y.-C., and Amon, A. (2013). Gene copy-number alterations: a cost-benefit analysis. Cell *152*, 394–405.

Teckchandani, A., and Cooper, J.A. (2016). The ubiquitin-proteasome system regulates focal adhesions at the leading edge of migrating cells. Elife *5*, https://doi.org/10.7554/eLife.17440.

The Gene Ontology Consortium (2015). Gene Ontology Consortium: going forward. Nucleic Acids Res. *43*, D1049–D1056.

The UniProt Consortium (2015). UniProt: a hub for protein information. Nucleic Acids Res. *43*, D204–D212.

Thompson, S.L., and Compton, D.A. (2011). Chromosomes and cancer cells. Chromosome Res. *19*, 433–444.

Wagner, S., Herrmannová, A., Malík, R., Peclinovská, L., and Valášek, L.S. (2014). Functional and biochemical characterization of human eukaryotic translation initiation factor 3 in living cells. Mol. Cell. Biol. *34*, 3041–3052.

Wang, J., Ma, Z., Carr, S.A., Mertins, P., Zhang, H., Zhang, Z., Chan, D.W., Ellis, M.J.C., Townsend, R.R., Smith, R.D., et al. (2016). Proteome profiling outperforms transcriptome profiling for co-expression based gene function prediction. Mol. Cell. Proteomics *16*, 121–134.

Xie, Y., Wolff, D.W., Wei, T., Wang, B., Deng, C., Kirui, J.K., Jiang, H., Qin, J., Abel, P.W., and Tu, Y. (2009). Breast cancer migration and invasion depend on proteasome degradation of regulator of G-protein signaling 4. Cancer Res. *69*, 5743–5751.

Ye, Y., Shibata, Y., Yun, C., Ron, D., and Rapoport, T.A. (2004). A membrane protein complex mediates retro-translocation from the ER lumen into the cytosol. Nature *429*, 841–847.

Zhang, B., Wang, J., Wang, X., Zhu, J., Liu, Q., Shi, Z., Chambers, M.C., Zimmerman, L.J., Shaddox, K.F., Kim, S., et al. (2014). Proteogenomic characterization of human colon and rectal cancer. Nature *513*, 382–387.

Zhang, H., Liu, T., Zhang, Z., Payne, S.H., Zhang, B., McDermott, J.E., Zhou, J.-Y., Petyuk, V.A., Chen, L., Ray, D., et al. (2016). Integrated proteogenomic characterization of human high-grade serous ovarian cancer. Cell *166*, 755–765.

Zolov, S.N., and Lupashin, V.V. (2005). Cog3p depletion blocks vesicle-mediated Golgi retrograde trafficking in HeLa cells. J. Cell Biol. *168*, 747–759.

OPEN ACCESS
CellPress

STAR★METHODS

KEY RESOURCES TABLE

REAGENT or RESOURCE	SOURCE	IDENTIFIER
Antibodies		
Mouse monoclonal anti-AP3B1	Abnova	Cat.#H00008546-B01P; RRID: AB_10714215
Rabbit monoclonal anti-AP3M1	Abcam	Cat.#ab201227; RRID: AB_2715538
Rabbit monoclonal anti-GTF2E1/TFIIEalpha	Abcam	Cat.#ab140634; RRID: AB_2715539
Rabbit monoclonal anti-GTF2E2/TFIIEbeta	Abcam	Cat.#ab187143; RRID: AB_2715540
Rabbit monoclonal anti-GAPDH(D15H11)	Cell Signaling Technologies	Cat.#5174S; RRID: AB_10622025
Goat-anti-rabbit IgG (HRP-linked)	Cell Signaling Technologies	Cat.#7074S; RRID: AB_2099233
Horse-anti-mouse IgG (HRP-linked)	Cell Signaling Technologies	Cat.#7076S; RRID: AB_330924
Bacterial and Virus Strains		
One Shot™ TOP10 Chemically Competent E. coli	Thermo Fisher	Cat.#C404003
Chemicals, Peptides, and Recombinant Proteins		
jetPEI transfection reagent	Polyplus transfection	Cat.#101-10N
Critical Commercial Assays		
DC™ protein assay	Bio-Rad	Cat.#500-0116
Deposited Data		
CPTAC proteomics of BRCA, HGSC and COREAD	CPTAC Consortium (Mertins et al., 2016; Zhang et al., 2016 and Zhang et al., 2014)	https://cptac-data-portal.georgetown.edu/cptacPublic/
TCGA transcriptomics RNA-seq raw counts	TCGA Consortium (Rahman et al., 2015)	GSE62944
TCGA copy-number GISTIC thresholded scores	TCGA Consortium (Mermel et al., 2011)	http://firebrowse.org/
Cell lines gene expression	Iorio et al., 2016b	E-MTAB-3610
Cell lines drug response	Iorio et al., 2016b	Table S4
Experimental Models: Cell Lines		
Human: HCT116	ATCC – LGC standards	Cat.# CCL-247; RRID: CVCL_0291
Human: HEK293	ATCC – LGC standards	Cat.# CRL-1573; RRID: CVCL_0045
Recombinant DNA		
psPAX2 – lentiviral packaging vector	Didier Trono Lab	Addgene plasmid #12260
pMD2.G – lentiviral Envelope vector	Didier Trono Lab	Addgene plasmid #12259
pLKO.1-shAP3B1 (TRCN0000286136) – shRNA	Sigma Aldrich	Cat.#SHCLND-NM_003664
pLKO.1-shAP3M1 (TRCN0000065101) – shRNA	Sigma Aldrich	Cat.#SHCLND-NM_012095
pLKO.1-shGTF2E1 (TRCN0000020722) – shRNA	Sigma Aldrich	Cat.#SHCLND-NM_005513
pLKO.1-shGTF2E2 (TRCN0000020775) – shRNA	Sigma Aldrich	Cat.#SHCLND-NM_002095
pLKO.1-shNT – shRNA	Sigma Aldrich	Cat.#SHC016-1EA
Software and Algorithms		
Quantity One® Basic software	Bio-Rad	N/A (Freeware)
GraphPad Prism 5.03 software	GraphPad	https://www.graphpad.com/
JMP® 10 software	SAS Institute Inc.	https://www.jmp.com/en_us/home.html
Limma	Ritchie et al., 2015	http://bioconductor.org/packages/release/bioc/html/limma.html
edgeR	Robinson et al., 2010	https://bioconductor.org/packages/release/bioc/html/edgeR.html
SLAPenrich	Iorio et al., 2016a	https://github.com/francescojm/SLAPenrich
Sklearn	Pedregosa et al., 2011	http://scikit-learn.org/

CONTACT FOR REAGENT AND RESOURCE SHARING

Further information and requests for resources and reagents should be directed to and will be fulfilled by the Lead Contact, Pedro Beltrao (pedrobeltrao@ebi.ac.uk).

EXPERIMENTAL MODEL AND SUBJECT DETAILS

The human colon cancer cell line HCT116 (male donor) was cultivated in McCoys 5a medium supplemented with 10% FBS and 1% penicillin/streptomycin under standard culture condition (at 37°C in a humidified 5% CO2 containing atmosphere). AP3B1, AP3M1, GTF2E1, and GTF2E2 silencing was obtained by lentiviral short hairpin RNA (shRNA) delivery. shNT (“non target”) clones were used as control cells. For protein sample isolation, 10^6 cells of shNT, shAP3B1, shAP3M1, shGTF2E1 or shGTF2E2 clones were plated in 10 cm culture dishes for 48h. Afterwards cells were lysed in RIPA buffer to obtain total protein samples. Protein content was determined by DC™ protein assay as recommended by the manufacturer (Bio-Rad laboratories Inc, Cat.#: 500-0116, Hercules, CA USA).

METHOD DETAILS

Cell Lines Drug Response Analysis

Gene expression measurements (E-MTAB-3610) acquired with Affymetrix Human Genome U219 array for approximately 1,000 cell lines was used in this analysis (Iorio et al., 2016b). Drug response measurements were obtained as the area under the curve (AUC) for 265 compounds (Iorio et al., 2016b). Cell lines proteome attenuation potential was calculated by performing pearson correlation between their transcriptomics profile and the proteome attenuation potential signature derived from tumours. Cell line correlations with the signature were then used as a feature in single linear regression models to systematically predict the response of each compound in the screen.

shRNA Delivery via Lentiviral Transduction

The applied shRNA plasmids (pLKO.1) are part of the MISSION® shRNA product line of Sigma Aldrich (shAP3B1, Cat.#: SHCLND-NM_003664, TRC clone: TRCN0000286136; shAP3M1, Cat.#: SHCLND-NM_012095, TRC clone: TRCN0000065101; shGTF2E1, Cat.#: SHCLND-NM_005513, TRC clone: TRCN0000020722; shGTF2E2, Cat.#: SHCLND-NM_002095, TRC clone: TRCN0000020775; shNT, Cat.#: SHC016-1EA) and were delivered via lentiviral transduction using a second generation lentiviral packaging system. Therefore, HEK293T cells were co-transfected with the appropriate pLKO.1 transfer-vector (shRNA containing vector), psPAX2 (the packaging vector, addgene #12260) and pMD2.G (the vector that encodes for the viral envelope protein, addgene #12259) using jetPEI transfection reagent according to manufacturer’s recommendation (Polyplus transfection, Cat.#: 101-10N, Illkirch, France). Virus-containing supernatants were used for cell transduction.

Western Blot Validation

Predicted protein complex formations of AP3B1_AP3M1 and GTF2E2_GTF2E1 were validated by western blot technique. Total protein lysates (30 μg) were heat-denatured in NuPAGE LDS sample buffer containing dithiothreitol (Thermo Scientific, Cat.#: NP0008, Waltham, MA USA) and loaded on 12% denaturing polyacrylamide gels for separation. SDS-PAGE was conducted with a 2-Step protocol (Step1: 20min 50V constant, Step2: 120min 120V constant). Proteins were transferred to nitrocellulose membranes by tank-blotting (140min at 70V constant). Afterwards membranes were blocked with 5% milk (MP) in TBS-T. All washing steps were conducted with TBS-T. Membranes were incubated with primary antibodies mc mouse α-AP3B1 (abnova, Cat.#: H00008546-B01P, Taipei City, Taiwan; 1:500), mc rabbit α-AP3M1 (abcam, Cat.#: ab201227, Cambridge, UK; 1:1000), mc rabbit α-GTF2E2/TFIIEbeta (abcam, Cat.#: ab187143, Cambridge, UK; 1:10000) or mc rabbit α-GTF2E1/TFIIEalpha (abcam, Cat.#: ab140634, Cambridge, UK; 1:1000) overnight at 4°C. Protein expression of GAPDH was used as loading control using α-GAPDH(D15H11) antibody (CST, Cat.#: 5174S, Cambridge, UK; 1:2000). All primary antibodies were diluted in 5% MP TBS-T. Secondary antibodies used in this work are: HRP-conjugated anti-rabbit IgG (CST, Cat.#: 7074S, Cambridge, UK) for the detection of AP3M1 (1:2000), GTF2E2 (1:1000), GTF2E1 (1:2000) & GAPDH (1:2000), and HRP-conjugated anti-mouse IgG (CST, Cat.#: 7076S, Cambridge, UK) for the detection of AP3B1 (1:5000). Secondary antibodies were diluted in TBS-T and incubated for 1h at room temperature. Quantity One® software (Bio-Rad laboratories Inc., Hercules, CA USA) was used for densitometry.

QUANTIFICATION AND STATISTICAL ANALYSIS

Data Compendium

Proteomics measurements at the protein level for the three tumour types analysed here were compiled from the CPTAC data portal (Edwards et al., 2015) (accession date 2016/07/06) for the following publications: BRCA (Mertins et al., 2016), HGSC (Zhang et al., 2016) and COREAD (Zhang et al., 2014). Transcriptomics RNA-seq raw counts were acquired from (Rahman et al., 2015) (GSE62944) and processed using the Limma R package (Ritchie et al., 2015) with the voom transformation (Law et al., 2014). GISTIC (Mermel et al., 2011) thresholded copy-number variation measurements and clinical data were obtained from the http://firebrowse.org/ portal (accession date 2016/06/08).

Data Processing and Normalisation

Transcriptomics raw counts were downloaded from (Rahman et al., 2015) (GSE62944). To ensure that lowly expressed transcripts are removed, genes with average counts per million (CPM) across samples lower or equal to 1 were excluded. Data was normalised by the trimmed mean of M-values (TMM) method (Robinson and Oshlack, 2010) using edgeR (Robinson et al., 2010) R package. Finally, the log-CPM values derived from the voom (Law et al., 2014) function in Limma (Ritchie et al., 2015) package were extracted for this analysis.

Coverage of the proteomics samples was assessed using the jaccard index for each sample with matching transcriptomics. Transcriptomics and proteomics measurements were used at the gene symbol level annotation. For each sample it was only considered transcripts passing the expression threshold, defined above, and proteins with matching measurement. The jaccard index for each sample was calculated with the intersection over the union.

Considering that proteomics and transcriptomics principal component analysis (PCA) revealed associations with possible confounding factors, i.e. age, gender, tumour type and measurement technology, we regressed them out from the original data-sets using linear regression models (Figure S1). For each protein a multiple linear regression model was fitted with protein measurements across the tumour samples as the dependent variable and the confounding factors mentioned above as independent discrete variables, apart from the age which was represented with a continuous variable. Once the model was fitted the estimated weights of the covariates were used to regress-out their impact in the protein measurement and thereby removing their effects (Figure S2). Due to the sparseness of mass-spectrometry measurements for the proteomics data-set we only considered proteins that were consistently measured in at least 50% of the samples, leaving a total of 6,734 proteins. The same procedure was performed in the transcriptomics measurements. Transcript and protein measurements were normalised and centered across the samples using a gaussian kernel density estimation function.

Proteome Attenuation Analysis

Agreement between the copy-number variation and the transcriptomics and proteomics was calculated for each gene/protein across the tumour samples using pearson correlation coefficient. Enrichment of biological processes for proteins displaying an attenuation of the correlation at the protein level compared to the transcript level was performed using Gene Set Enrichment Analysis (GSEA) (Subramanian et al., 2005). For the enrichment we used the protein attenuation level, which is calculated by the difference between the pearson coefficient of the transcript correlation (correlation between copy-number variation and transcript measurements) and the pearson coefficient of the protein correlation (correlation between copy-number and protein measurements). To ensure a normal distribution centered around zero for the GSEA enrichments a gaussian kernel density estimation function was used to normalise the protein attenuation distribution. Gene signatures of Gene Ontology (GO) (Ashburner et al., 2000; The Gene Ontology Consortium, 2015) terms for biological processes (BP) and cellular compartments (CC) were acquired from the MSigDB data-base (Subramanian et al., 2005). Gene signatures of post-translational modifications (PTMs) were also used and acquired from Uniprot data-base (The UniProt Consortium, 2015). The estimated enrichment scores were statistically assessed by performing 1,000 random permutations of the signatures and p-values were then adjusted using false-discovery rate (FDR).

Proteins were classified according to their copy-number attenuation effect using a gaussian mixture model with 2 mixture components. Proteins in the group with larger mean attenuation were considered highly attenuated. More stringent classification of the attenuation effect was performed by only considering attenuated proteins with an absolute attenuation score higher than 0.3.

For samples the attenuation potential was estimated similarly as for proteins but instead correlations were calculated across the proteins measured in the sample. Samples were then classified as before with a gaussian mixture model with 2 mixture components. Enrichment analysis of amplifications in protein complexes in tumour samples with high protein attenuation potential was performed using SLAPenrich (Iorio et al., 2016a).

A gene expression signature of the sample attenuation potential was calculated by systematically correlating the samples attenuation potential with each gene in the transcriptomics data-set.

Pairwise Correlation Analysis

Correlations between protein pairs, or genes, across samples were calculated using pearson correlation coefficient. Only proteins that were also measured at the transcript level were considered, i.e. 6,434. The systematic analysis of all unique pairwise correlations generated a total of 41,389,922 correlation coefficients both at the protein and gene level.

Protein sets of known protein complexes were acquired from the CORUM data-base (Ruepp et al., 2010, 2008). A protein-protein interaction list of the complexes was assembled by considering that two proteins interact if they are present within the same complex at least once, this generated a total of 67,927 interactions. Indirect but functional associations were also considered by using the STRING data-base (Franceschini et al., 2013). For STRING only interactions with the highest confidence score (900) were used performing a total of 214,815 interactions. 9,273 protein interactions within signalling pathways were assembled from kinase/phosphatase-substrate interactions reported in SIGNOR data-base (Perfetto et al., 2016). Metabolic enzyme interactions associated with metabolic pathways were extracted from KEGG pathways (Kanehisa et al., 2016) reported in MSigDB (Subramanian et al., 2005). Two enzymes were considered to be interacting if they were present in the same metabolic pathway, making a total of 121,134 interactions. Enrichment of the different types of protein-protein interactions, i.e. complexes, functional, signalling and metabolic, were estimated using receiving operating characteristic (ROC) curves and by calculating the area under the ROC curve (AROC). True-positive sets of protein interactions were defined as the ones reported in the different resources used. Due to the strong unbalance between

the number of true positives and false positives the ROC curves were calculated using 5 different and randomised sets within the false positive group. The variability of the AROC score is represented by error bars in Figure 3C.

Proteogenomics Analysis to Identify Protein Complex Regulators

The identification of protein complex regulators only focused on protein-protein interactions reported in the CORUM data-base (Ruepp et al., 2010, 2008) with a protein-protein interaction list assembled as described before.

For each protein-protein interaction reported within a complex, its association was tested using two linear regression models. Given a pair Protein Y ~ Protein X (Py ~ Px), a first linear model is used to regress-out the transcript variability from the protein measurement of Py. The dependent variable of the model is the proteomics measurements of Py and the independent variable is the transcriptomics measurements (Ty) (Equation 1):

$$Py = \beta.\ Ty + \psi \qquad \text{(Equation 1)}$$

The model is fit with an intercept (for simplicity omitted from Equation 1) and noise term, ψ. After fitting the estimated weight (β), the residuals of Py (Py') are calculated as (Equation 2):

$$Py' = Py - \beta.\ Ty \qquad \text{(Equation 2)}$$

Py' represents the variability measured due to post-transcriptional and post-translational regulation. Then a second linear model is performed to calculate the association between Py' and the CNV of Px, (Px) (Equation 3):

$$Py' = \beta.\ Px + \psi \qquad \text{(Equation 3)}$$

Statistical significance is estimated by calculating an F statistic over an F-distribution, p-values are then adjusted using FDR correction. A total of 58,627 tests are performed. The same analysis is performed using transcriptomics measurements instead of the copy-number of Px, generating a total of 57,462 tests. Associations estimated with the copy-number variation that are significant with the transcriptomics are highlighted with a red border in Figure 3A.

Logistic Classification of Samples Protein Attenuation Potential

The predictive power of the attenuation potential gene expression signature was benchmarked using logistic classification models. This was performed using 1,000 randomised groups of 30% of samples for testing and 70% for training. Feature selection was performed using ANOVA F-value following by FDR multiple hypothesis correction and features with FDR lower than 5% were kept for training and testing. The regularisation term of the logistic classification models were optimised using a stratified cross-validation approach.

Statistical Analysis of Experimental Data

Variance homogeneity was checked with the Bartlett test. The Shapiro-Wilk test was used to test normal distribution. The two-tailed unpaired t-test was applied to analyze differences between shNT and the corresponding knockdown group. Data are shown as mean + SEM, n = 3 (3 independent experiments). * $p < 0.05$ vs. shNT. Analyses were carried out with GraphPad Prism 5.03 (GraphPad, La Jolla, USA) and JMP 10 (Böblingen, Germany).

Code Availability

All the computational analyses were performed in Python version 2.7.10, apart from the transcriptomics RNA-seq processing which as done in R version 3.3.1 with Limma package version 3.28.21 and edgeR 3.14.0, and are available under GNU General Public License V3 in a GitHub project in the following url https://github.com/saezlab/protein_attenuation. Plotting was done using Python modules Matplotlib version 1.4.3 (Hunter, 2007) and Seaborn version 0.7.0. Generalised linear models were built using Python module Sklearn version 0.17.1 (Pedregosa et al., 2011). Data analysis and structuring was carried out using Python module Pandas version 0.18.1 (McKinney, 2010).

Cell Systems, Volume *5*

Supplemental Information

Widespread Post-transcriptional Attenuation of Genomic Copy-Number Variation in Cancer

Emanuel Gonçalves, Athanassios Fragoulis, Luz Garcia-Alonso, Thorsten Cramer, Julio Saez-Rodriguez, and Pedro Beltrao

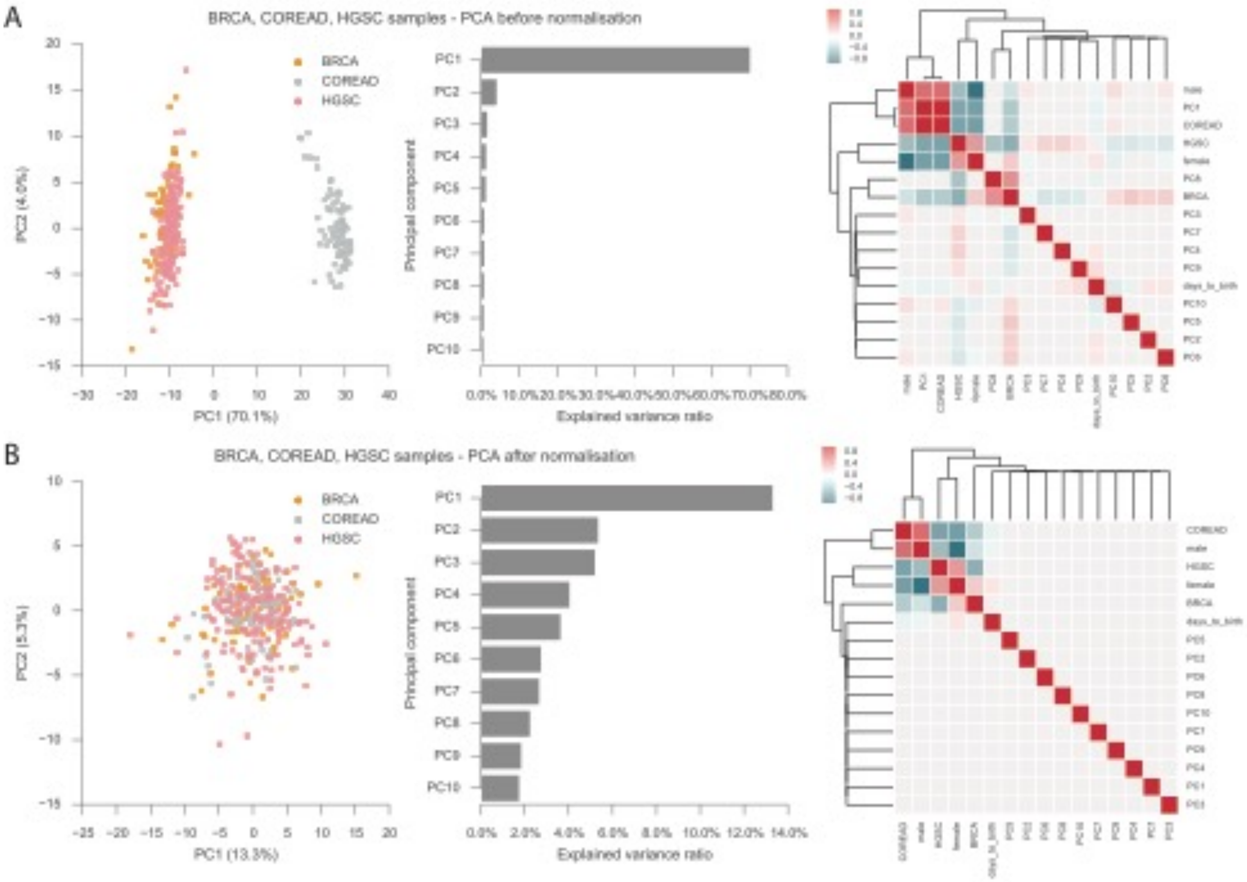

Supplementary Figure 1. Related to Figure 1. Proteomics data-sets PCA analysis using proteins consistently measured across all the samples and pearson correlation coefficient between the first 10 principal components and the possible confounding factors, i.e. age, tumour type and gender. A) Analysis performed on the original proteomics data-sets. B) Analysis performed on the proteomics data-set after the confounding factors were regressed-out.

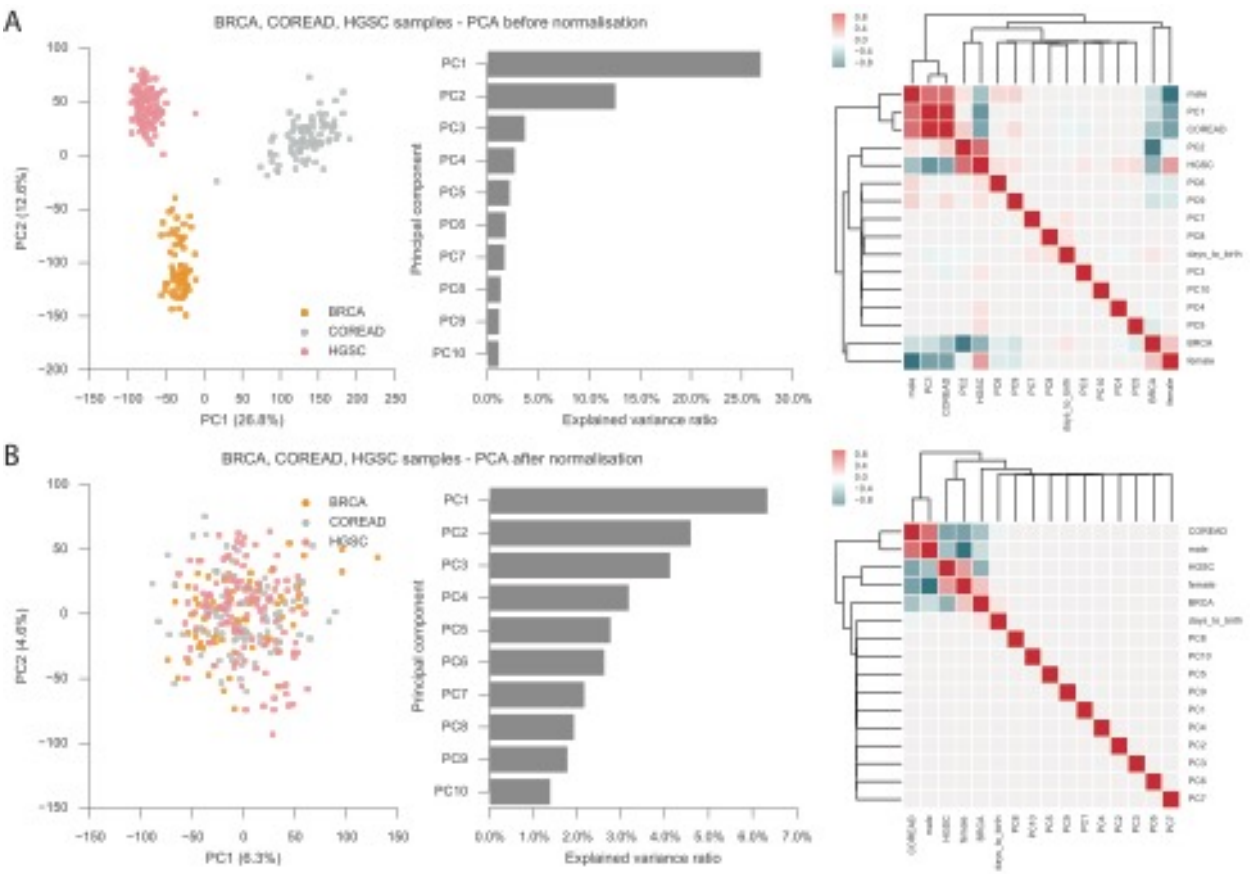

Supplementary Figure 2. Related to Figure 1. Transcriptomics data-sets PCA analysis and pearson correlation coefficient between the first 10 principal components and the possible confounding factors, i.e. age, tumour type and gender. A) Analysis performed on the voom transformed transcriptomics measurements. B) Analysis performed on the transcriptomics data-set after the confounding factors were regressed-out.

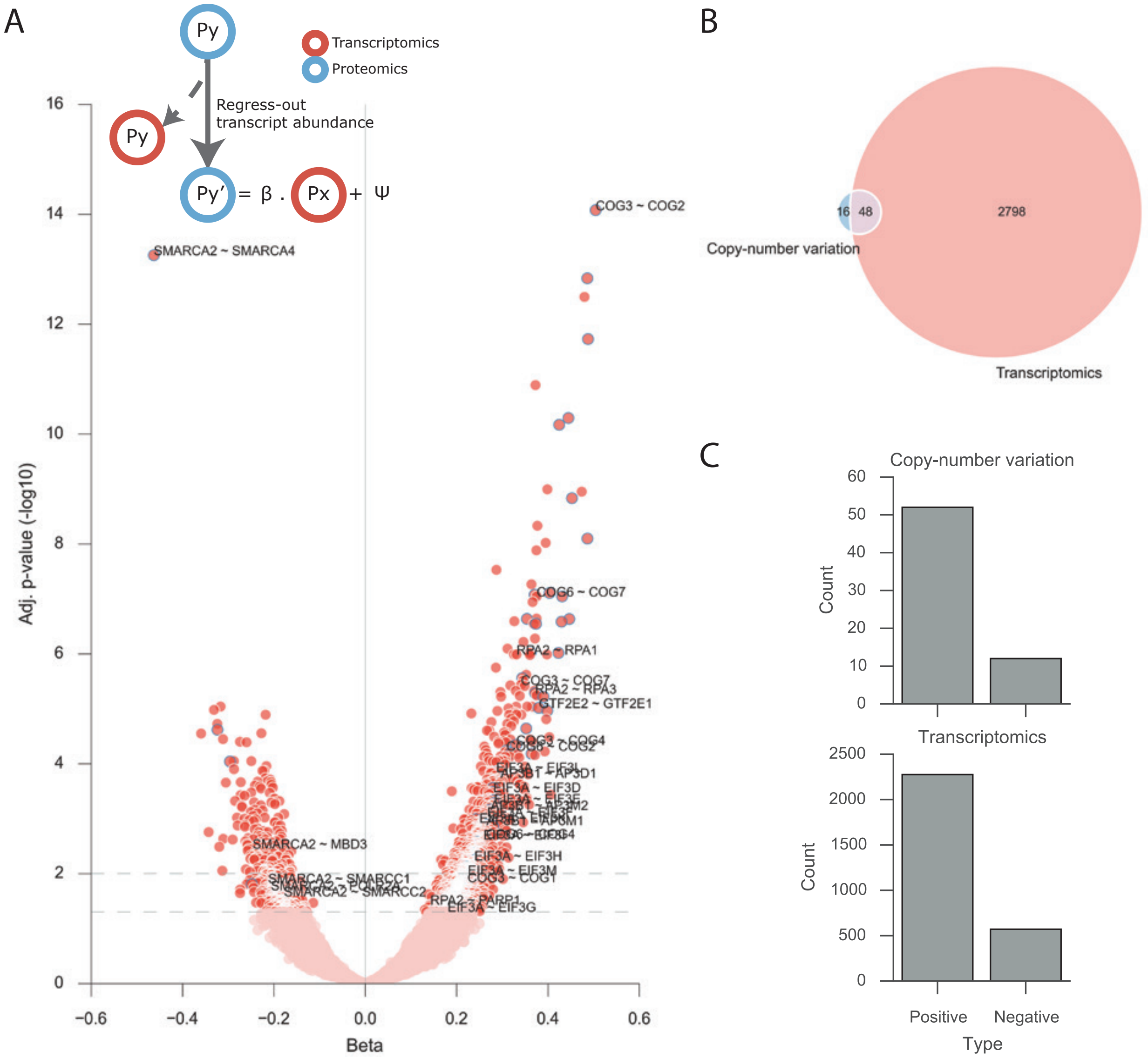

Supplementary Figure 3. Related to Figure 3. Protein complexes regulatory interactions identified using transcriptomics of the putative regulatory protein (Px). A) Volcano plot representing the effect size on the x axis and FDR adjusted p-value on the y axis. Diagram representing the linear model used to perform the associations. B) Overlap between the significant regulatory associations found using the copy-number variation and transcriptomics of the Px proteins. C) Number of significant associations with a Positive or Negative effect size.

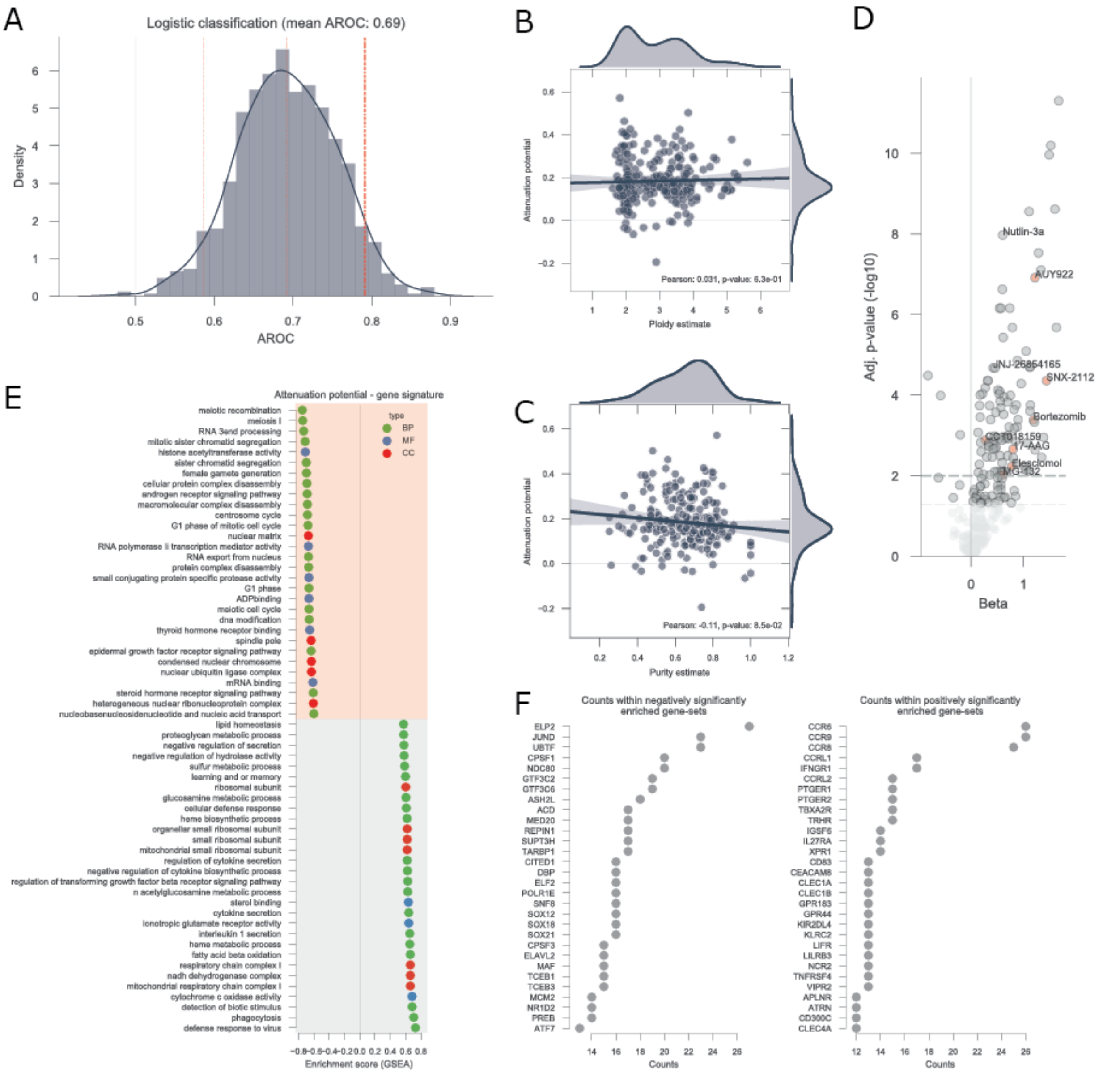

Supplementary Figure 4. Related to Figure 7. Tumour and cell lines samples attenuation potential analysis. A) Benchmark of the gene-signature protein attenuation potential of the tumour samples using a logistic classification model. B) & C) Tumour samples attenuation potential correlation with ploidy and purity, respectively. D) Volcano plot of the drug response associations performed in the cell line panel using the cell lines putative attenuation potential as the predictive feature. Significant associations (FDR < 5%) of chaperone and proteasome inhibitors are labelled and marked in red. Ubiquitin-protein ligase MDM2 inhibitors are labelled. E) GO terms enrichment analysis of the protein attenuation gene-expression signature. Top 30 positive and negative significantly enriched GO terms are shown. Red background denotes GO terms enriched for genes correlating positively with the protein attenuation potential, blue denotes enrichments for negative correlations. F) Shows the genes more frequently present in the significantly enriched gene-signatures. Top 30 most frequent genes are shown for the negative (left) and positive (right) enriched gene-sets.
